# Long noncoding RNA NONMMUT015745 inhibits doxorubicin-mediated cardiomyocyte apoptosis by regulating Rab2A-p53 axis

**DOI:** 10.1038/s41420-022-01144-9

**Published:** 2022-08-16

**Authors:** Hongjing Cai, Pengchao Tian, Jie Ju, Tao Wang, Xinzhe Chen, Kai Wang, Fei Wang, Xue Yu, Shaocong Wang, Yin Wang, Chan Shan, Peifeng Li

**Affiliations:** 1 Institute of Translational Medicine, The Affiliated Hospital of Qingdao University, College of Medicine, Qingdao University, Qingdao, China; 2 Department of Pathophysiology, Binzhou Medical University, Yantai, China; 3 State Key Laboratory of Cardiovascular Disease, Heart Failure center, Fuwai Hospital, National Center for Cardiovascular Diseases, Chinese Academy of Medical Sciences, Peking Union Medical College, Beijing, China

**Keywords:** Long non-coding RNAs, Heart failure, Apoptosis

## Abstract

Doxorubicin (DOX) is an efficacious and widely used drug for human malignancy treatment, but its clinical application is limited due to side effects, especially cardiotoxicity. Our present study revealed that DOX could induce apoptosis in cardiomyocytes. Herein, we screened the dysregulated long noncoding RNAs (lncRNAs) in DOX-treated cardiomyocytes. Notably, overexpression of lncRNA NONMMUT015745 (lnc5745) could alleviate DOX-induced cardiomyocyte apoptosis both in vitro and in vivo. Conversely, silencing lnc5745 promotes cardiomyocyte apoptosis. Moreover, Rab2A, a direct target of lnc5745, possesses a protective effect in DOX-induced cardiotoxicity once knocked down. Importantly, we verified that the p53-related apoptotic signalling pathway was responsible for the lnc5745-mediated protective role against DOX-induced cardiomyocyte apoptosis. Mechanistically, Rab2A interacts with p53 and phosphorylated p53 on Ser 33 (p53 (Phospho-Ser 33)), promotes p53 phosphorylation, thereby activating the apoptotic pathway. Taken together, our results suggested that lnc5745 protects against DOX-induced cardiomyocyte apoptosis through suppressing Rab2A expression, modifying p53 phosphorylation, thereby regulating p53-related apoptotic signalling pathway. Our findings establish the functional mode of the lnc5745-Rab2A-p53 axis in DOX-induced cardiotoxicity. The development of new strategies targeting the lnc5745-Rab2A-p53 axis could attenuate DOX-induced cardiotoxicity, which is beneficial to its clinical anti-tumour application.

## Introduction

Doxorubicin (DOX), also known as Adriamycin, is a first-line anthracycline group of antibiotics that has been widely used as an efficacious chemotherapeutic for human malignancies, including solid sarcomas, breast carcinomas, haematological malignancies and soft tissue sarcomas, since the late 1960s [1, 2]. However, the clinical use of DOX is limited due to severe dose-dependent and cumulative cardiotoxicity [3]. Increasing the dosage of DOX will cause irreversible myocardial damage and ultimately lead to dilated cardiomyopathy (DCM) and congestive heart failure [4, 5]. Therefore, a thorough comprehension of the pathogenesis of DOX cardiomyopathy and the recognition of novel therapeutic targets for protecting cardiac function are urgently needed.

Recent studies have shown that the molecular mechanism responsible for the cardiotoxicity of DOX appears to be multifactorial [6]. Excessive ROS can induce oxidative damage to biological macromolecules, including DNA, proteins and lipids, and destroy the integrity and function of cell membranes [7–9]. Moreover, DOX-induced oxidative stress can directly induce cardiomyocyte apoptosis through multiple apoptotic pathways, leading to severe cardiac dysfunction. In addition, DOX can also evoke apoptosis in a non-ROS- and oxidative stress-dependent manner [10]. Our recent studies have demonstrated that inhibition of cardiomyocyte apoptosis can significantly attenuate DOX-induced cardiac dysfunction [11]. Therefore, a thorough comprehension of the pathogenesis of DOX-induced cardiomyocyte apoptosis and the recognition of novel therapeutic targets for protecting cardiac function are urgently needed.

Long noncoding RNAs (lncRNAs) are an important class of noncoding transcripts more than 200 nucleotides in length that lack functional open reading frames [12, 13] and thus do not code for proteins [14]. Increasing evidence shows that lncRNAs participate in the regulation of life events through multiple mechanisms, including epigenetic regulation, genomic imprinting, protein modification, RNA stability and RNA alternative splicing [15, 16]. LncRNAs are involved in the regulation of cardiac functions, such as cardiac growth and morphogenesis [17], electrical signal propagation and myocardial contraction [18, 19]. Previous findings have clarified that lncRNAs are powerful controllers of DCM, heart failure (HF) and myocardial infarction (MI) by regulating cardiomyocyte necrosis, necroptosis, and autophagy [20]. However, detailed studies on the influence of lncRNAs in regulating DOX-induced cardiomyocyte apoptosis are still limited.

p53 is widely known as a transcription factor and a tumour suppressor that possesses various functional roles in cells by regulating multiple regulatory signals to ensure sufficient time and space to respond to cellular stress [21]. Among these pathways, the p53 apoptotic pathway occupies an important position. The p53 apoptotic pathway is activated under external stress or DNA damage. Upon activation, acetylated p53 migrates to the mitochondria, where it regulates outer mitochondrial membrane (OMM) permeabilization through interactions with BCL-2 family members such as BAX, BAK, BCL-2, and BCL-XL [22–25]. Once OMM permeability increases, apoptotic effectors such as cytochrome C are released into the cytoplasm from mitochondria [26] and they trigger the activation of a series of caspase cascades and the cleavage of PARP, which ultimately promotes cell apoptosis [27]. Although the role of the p53-related apoptosis pathway in doxorubicin cardiotoxicity has been investigated [28–31], it has not yet been linked to lncRNAs.

Our present study investigated the molecular mechanism underlying DOX-induced cardiomyopathy. LncRNA NONMMUT015745 (hereafter referred to as lnc5745) was significantly downregulated in neonatal mouse ventricular cells (NMVCs) and a mouse model under DOX treatment. Overexpression of lnc5745 protects cardiomyocytes from apoptosis and cell death induced by DOX treatment. Specifically, DOX cardiotoxicity was alleviated in adenovirus-mediated lnc5745-overexpressing mice, and cardiac function was improved. Rab2A, a direct target of lnc5745, can be suppressed by lnc5745 through the proteasome pathway. Additionally, we verified that Rab2A silencing could alleviate DOX-induced cardiomyocyte apoptosis. Mechanistically, Rab2A could directly interact with p53 and p53 (Phospho-Ser 33), increase p53 phosphorylation, thereby activating the apoptotic pathway, and promoting cardiomyocyte apoptosis subsequently. Our research demonstrated that the lnc5745-Rab2A axis defines a novel anti-apoptotic signal to alleviate DOX-induced cardiotoxicity, providing new insights and strategies for avoiding DOX cardiotoxicity during clinical applications.

## Results

### Lnc5745 was identified, and its downregulation in the NMVCs was correlated with DOX treatment

**Fig. 1:**
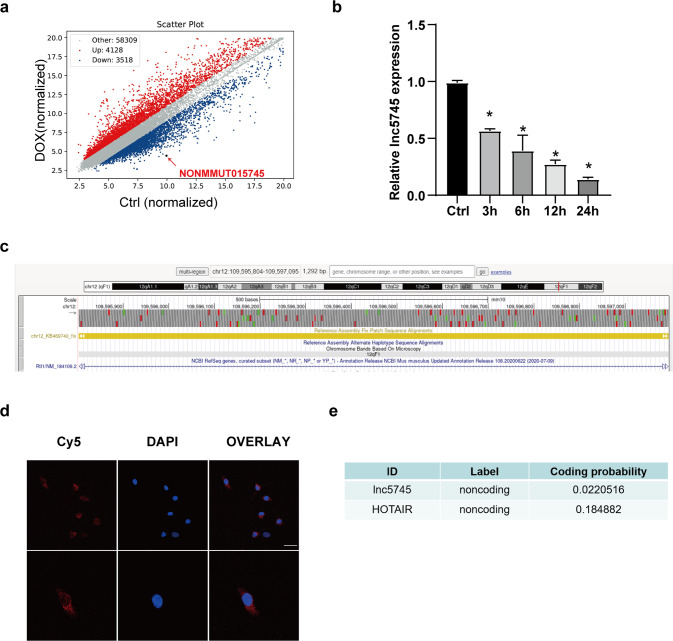
Lnc5745 is downregulated in NMVCs treated with DOX. **a** Microarray results showing the differentially expressed lncRNAs in NMVCs untreated and treated with DOX. The red and blue dots represent the lncRNAs that are upregulated and downregulated more than 2.5-fold, respectively. The red arrow points to lnc5745. **b** Expression of lnc5745 was downregulated in NMVCs after 1 μM DOX treatment for 3, 6, 12 and 24 hours. **c** UCSC Genome Browser view of lnc5745. **d** Fluorescence in situ hybridization (FISH) showing the localization and expression of lnc5745 in NMVCs. Lnc5745 was captured by specific Cy5-labelled probes (red). Nuclei were stained by DAPI (blue). Scale bar, 25 μm. **e** CPC (coding potential calculator) 2.0 predicts that lnc5745 possesses a very low coding probability, similar to HOTAIR. The results are from three independent experiments, *P < 0.05. Variables are presented as the mean ± SD.

To explore the specific mechanisms and influencing factors of DOX-induced cardiomyopathy, we performed lncRNA microarray analysis in NMVCs untreated and treated with DOX. A total of 7646 lncRNAs were represented, among which 4128 were upregulated and 3518 were downregulated when filtered using a threshold of a fold-change ≥ 2.5 and *P* < 0.05 (Fig. 1a). Then, the ten most significantly downregulated lncRNAs were verified by qRT–PCR (Supplementary Fig. S1a). Lnc5745 was stable and significantly downregulated in time-dependent manner in NMVCs after DOX (1 μM) treatment compared with the control group (Fig. 1b).

Lnc5745 is an intergenic lncRNA located on chromosome 12qF1, with no exons and a full length of 1292 nucleotides (Fig. 1c). To determine the subcellular localization of lnc5745, we employed FISH analysis. The results revealed that lnc5745 was mainly distributed in the cytoplasm (Fig. 1d). The CPC (coding potential calculator) 2.0 computational algorithm [32] predicted that lnc5745 possesses a very low coding probability, similar to HOTAIR, a well-known lncRNA (Fig. 1e). Therefore, lnc5745 is downregulated upon DOX exposure, and its role in DOX-induced cardiac injury needs further study.

### Lnc5745 attenuated apoptosis in cardiomyocytes treated with DOX

**Fig. 2:**
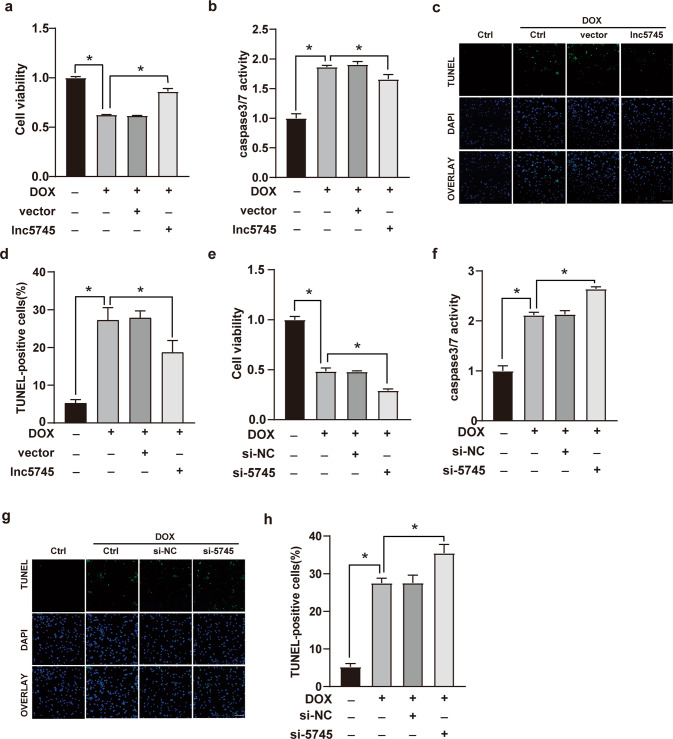
Lnc5745 inhibits DOX-induced cardiomyocyte apoptosis in NMVCs. **a** CCK-8 assays showing that lnc5745 overexpression increased NMVC viability under DOX treatment. **b** Lnc5745 inhibited caspase-3/7 activation stimulated by DOX in NMVCs. **c** Apoptotic cells were observed with the TUNEL method (green). The nuclei were stained with DAPI (blue). Scale bar, 200 μm. Lnc5745 overexpression decreased cell apoptosis induced by DOX. **d** Ratio of TUNEL-positive cardiomyocytes was determined by ImageJ (*n* = 200). **e** Lnc5745 knockdown reduced NMVC viability under DOX treatment. **f** Lnc5745 knockdown increased DOX-stimulated caspase-3/7 activity. **g**, **h** Apoptotic cells were observed with TUNEL method (green). Nucleus were stained by DAPI (blue). Lnc5745 knockdown further increased DOX-induced cardiomyocyte apoptosis. Scale bar, 200 μm. The results are from three independent experiments, *P < 0.05. Variables are presented as the mean ± SD.

To clarify the potential role of lnc5745 in DOX-induced cardiotoxicity, NMVCs were transfected with a pCDNA3.1-lnc5745 vector. Real-time PCR showed that the lnc5745 level increased over 20-fold compared with the negative control (Supplementary Fig. S1b). Overexpression of lnc5745 had no obvious effect on the viability and apoptosis rate of NMVCs (Supplementary Fig. S1c, d, e) but reduced caspase-3/7 activity (Supplementary Fig. S1f). Overexpression of lnc5745 significantly alleviated DOX-induced myocardial cell death (Fig. 2a) and apoptosis (Fig. 2b, c, d) after exposure to 1 μM DOX. Conversely, we suppressed endogenous lnc5745 using a lnc5745 siRNA (si-5745) to mimic the downregulation of lnc5745 by DOX treatment (Supplementary Fig. S1g). siRNA mediated lnc5745 downregulation reduced the viability (Supplementary Fig. S1h) and promoted apoptosis (Supplementary Fig. S1i–k) of NMVCs. Knockdown of lnc5745 aggravated the cardiotoxicity induced by DOX treatment, which was manifested by increased cell death (Fig. 2e) and apoptosis (Fig. 2f–h) of cardiomyocytes. Altogether, these results suggested a protective effect of lnc5745 on DOX-induced apoptosis in cardiomyocytes.

### Lnc5745 attenuated DOX-induced cardiac dysfunction in mice

**Fig. 3:**
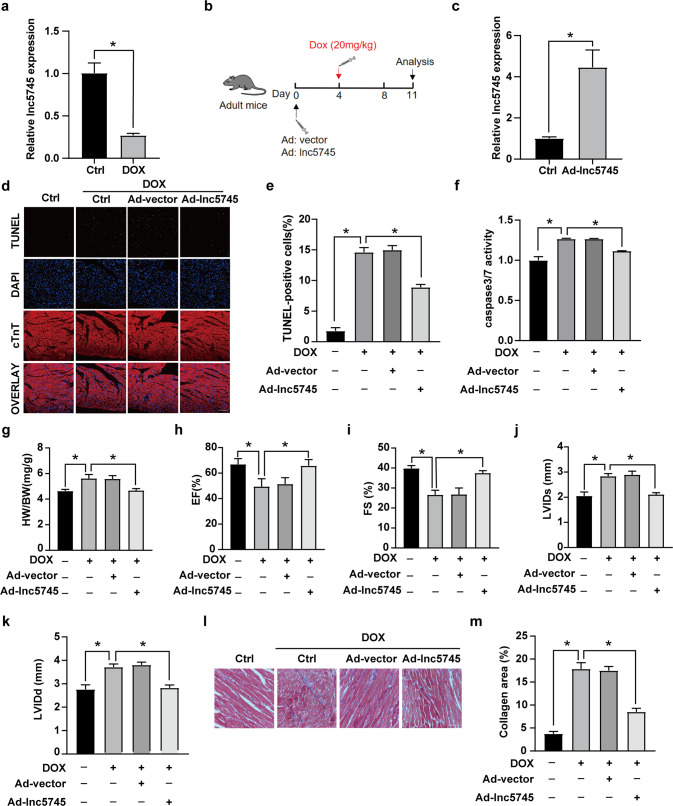
Lnc5745 improves cardiac function in DOX-induced cardiomyopathy mice. **a** Expression of lnc5745 was downregulated in mouse heart tissues after exposure to 20 mg/kg DOX for 7 days. **b** Flow chart of animal experiment. **c** Lnc5745 levels were significantly upregulated in the myocardial tissues of mice injected with adenovirus lnc5745 (Ad-lnc5745). **d** Apoptotic cells were observed with the TUNEL method (green). Nuclei were stained by DAPI (blue). Cardiac troponin T was captured by a specific antibody (red). Scale bar, 200 μm. Lnc5745 overexpression decreased cell apoptosis induced by DOX. **e** Ratio of TUNEL-positive cardiomyocytes in mice was counted by ImageJ (*n* = 200). **f** Lnc5745 inhibited caspase-3/7 activity elevation stimulated by DOX in mice. **g** Lnc5745 inhibited the DOX-induced increase in the heart-to-body weight ratio in mice. **h**–**k** Echocardiographic analysis revealed that lnc5745 improved cardiac function in the mouse model. **l** Representative images showing Masson trichrome staining for collagen. Scale bars, 50 µm. **m** Quantitative analysis of the collagen area after Masson trichrome staining. *n* = 6 per group, *P < 0.05. Variables are presented as the mean ± SD.

DOX treatment caused prominent cardiomyocyte apoptosis. Due to the limited regenerative capacity of cardiomyocytes, the loss of cardiomyocytes cannot be supplemented. Then, cardiomyocytes suffer compensatory hypertrophy, leading to dilated cardiac hypertrophy and decreased heart function under DOX treatment [33]. In a mouse model exposed to a single injection of 20 mg/kg DOX, lnc5745 expression was markedly decreased in heart tissues compared with the solvent control (Fig. 3a). To investigate the function of lnc5745 in DOX cardiotoxicity, adenovirus-mediated overexpression of lnc5745 was induced in murine heart tissues (Fig. 3b). Real-time PCR revealed that lnc5745 was over 4-fold upregulated in the mouse model (Fig. 3c). Overexpression of lnc5745 significantly reduced DOX-induced myocardial cell apoptosis (Fig. 3d–f). We measured the heart-to-body weight ratio to examine the role of lnc5745 in DOX-induced cardiac hypertrophy. The heart-to-body weight ratio was increased in mice treated with DOX, while the increase was significantly inhibited in the lnc5745-overexpressing group (Fig. 3g). In addition, we examined the effect of lnc5745 on cardiac function using echocardiography analysis. Cardiac function was significantly improved in lnc5745-overexpressing mice compared with the negative control (Fig. 3h–k). Moreover, in the histology test results, increased interstitial fibrosis occurred after DOX exposure, which could be suppressed by the overexpression of lnc5745 (Fig. 3l, m). In conclusion, lnc5745 effectively reduced DOX-induced cardiotoxicity and significantly improved cardiac function in a mouse model.

### Rab2A was a target of lnc5745

**Fig. 4:**
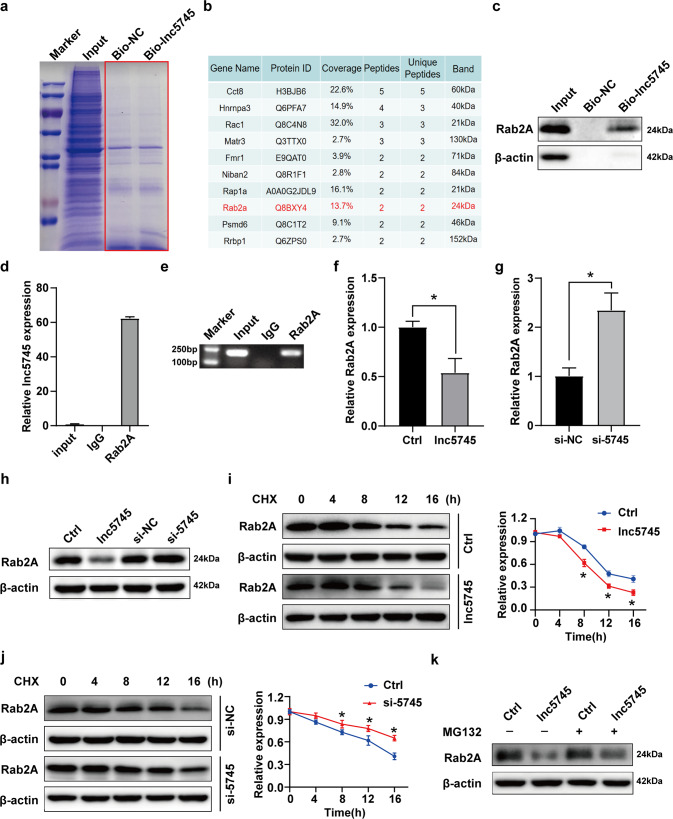
Lnc5745 targets Rab2A. **a** RNA pulldown assays were performed, and the lnc5745-related proteins were determined with SDS-polyacrylamide gel electrophoresis (SDS–PAGE) and Coomassie brilliant blue staining. **b** List of lnc5745-related proteins identified by mass spectrometry (MS). **c** Western blot analysis confirmed the existence of Rab2A in lnc5745-related proteins. **d**, **e** Binding of Rab2A to lnc5745 was confirmed by qPCR and PCR after RNA immunoprecipitation (RIP). **f**–**h** Rab2A levels were negatively affected by lnc5745 in NMVCs. **i** Effect of lnc5745 overexpression on Rab2A expression after CHX treatment for 0, 4, 8, 12 and 16 hours. **j** Effect of lnc5745 knockdown on Rab2A expression after CHX treatment for 0, 4, 8, 12 and 16 hours. **k** Effect of MG132 treatment for 12 hours on the stability of Rab2A protein. The results are from three independent experiments, *P < 0.05. Variables are presented as the mean ± SD.

It has been proved that lncRNAs exert their biological functions mainly by interacting with RNA-binding proteins [34]. We analysed the functional proteins that interact with lnc5745 to clarify the molecular mechanisms by which lnc5745 regulates cardiomyocyte apoptosis. RNA pulldown assays were performed by using a 3’-biotinylated lnc5745 probe or a 3’-biotinylated negative control probe (a randomly scrambled sequences based on lnc5745 probe) with cardiomyocyte lysates (Fig. 4a). Mass spectrometry (MS) was then employed to identify coprecipitated proteins as candidates for the lnc5745 interaction on a Q Exactive (QE) mass spectrometer (Fig. 4b). Among these, we focused on Rab2A. RAb2A is a member of the Rab family, which encodes a small guanosine triphosphatase (GTPase) protein and is necessary for ER-to-Golgi transport [35]. Importantly, RNA pulldown followed by western blot confirmed that lnc5745 could directly bind with Rab2A in NMVCs (Fig. 4c). In addition, RNA immunoprecipitation (RIP) assays followed by qRT-PCR and PCR revealed that the anti-Rab2A antibody effectively enriched lnc5745 (Fig. 4d, e). Next, we investigated the influence of lnc5745 on the expression and stability of Rab2A. Real-time PCR showed that overexpression of lnc5745 inhibited the transcription of Rab2A, while knockdown of lnc5745 by siRNA increased the Rab2A transcription level (Fig. 4f, g). Additionally, the western blot results confirmed the negative regulatory effect of lnc5745 on Rab2A at the protein expression level (Fig. 4h). Then we investigated how lnc5745 regulates Rab2A expression in NMVCs. Under the treatment of the protein synthesis inhibitor cycloheximide (CHX), the expression of Rab2A further decreased after lnc5745 overexpression, while increased significantly in the si-5745 group (Fig. 4i, j). Moreover, the proteasome inhibitor MG-132 treatment restored the lnc5745 overexpression-induced Rab2A downregulation in NMVCs (Fig. 4k). However, the expression of Rab2A did not change significantly after the lysosome inhibitor NH_4_Cl treatment (Supplementary Fig. S2a). Altogether, these results suggest that lnc5745 directly interacts with Rab2A and decreases the stability of Rab2A protein through the proteasome pathway.

### Rab2A participated in the regulation of DOX-induced cardiomyocyte apoptosis

**Fig. 5:**
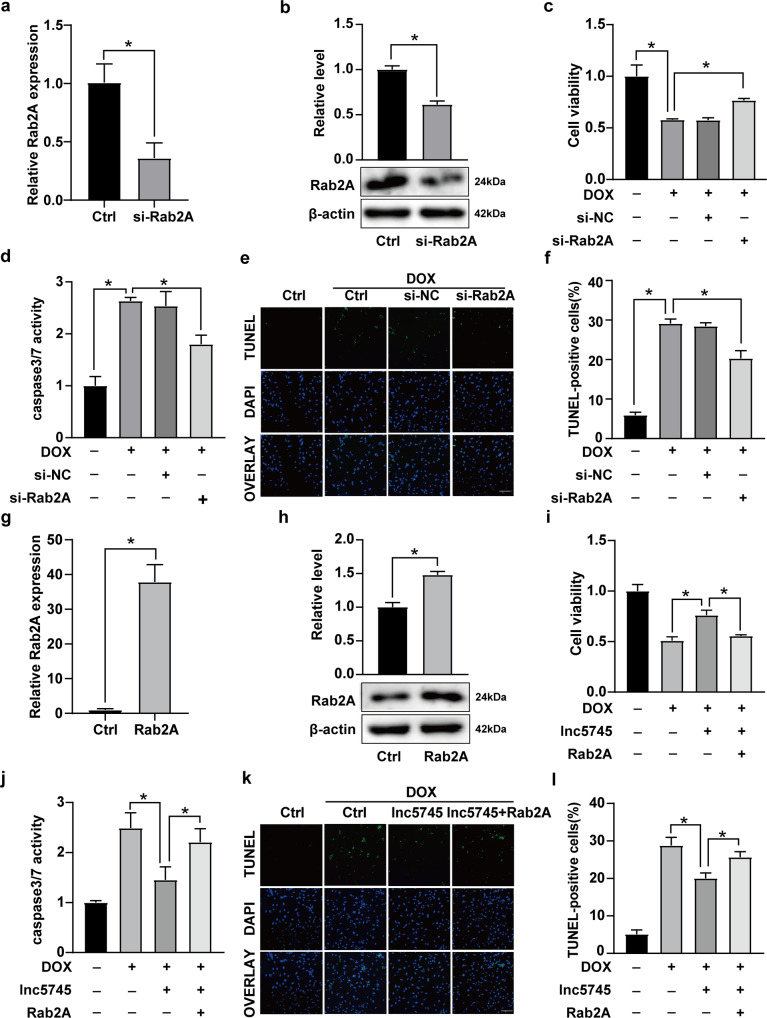
Rab2A alleviates the protective effect of lnc5745 on DOX-induced apoptosis. **a**, **b** Rab2A levels were significantly downregulated in NMVCs 24 h after transfection with Rab2A siRNA (si-Rab2A). **c** Rab2A knockdown increased NMVC viability under DOX treatment. **d**–**f** Rab2A knockdown reduced DOX-induced cardiomyocyte apoptosis. Scale bar, 200 μm. **g**, **h** Rab2A levels were significantly upregulated in NMVCs transfected with pCDNA3.1-Rab2A vectors. **i** Rab2A overexpression attenuated the lnc5745-induced vitality decrease in cardiomyocytes. **j**–**l** Rab2A overexpression attenuated the effect of lnc5745 on cardiomyocyte apoptosis. Scale bar, 200 μm. The results are from three independent experiments, *P < 0.05. Variables are presented as the mean ± SD.

To investigate the function of Rab2A in DOX cardiotoxicity, we inhibited the expression of Rab2A using Rab2A siRNA (si-Rab2A). The results of real-time PCR and western blot analysis showed that si-Rab2A significantly suppressed the expression of Rab2A (Fig. 5a, b). Knockdown of Rab2A alleviated the cardiotoxicity induced by DOX treatment, which was manifested by decreased cell death (Fig. 5c) and apoptosis (Fig. 5d–f) of cardiomyocytes. These results indicate that Rab2A participates in the regulation of DOX-induced cardiotoxicity.

### Lnc5745 alleviated DOX-induced apoptosis in cardiomyocytes by targeting Rab2A

We then examined whether lnc5745 attenuated cardiomyocyte apoptosis by targeting Rab2A. Transfection-mediated overexpression of Rab2A (Fig. 5g, h) attenuated the protective effect of lnc5745 overexpression against DOX-induced cardiotoxicity (Fig. 5i–l). In other words, the protective role of lnc5745 upon DOX-induced cardiotoxicity is lost by Rab2A overexpression. These data reveal that lnc5745 alleviated DOX-induced cardiomyocyte apoptosis by targeting Rab2A.

### p53 apoptotic signalling was responsible for the lnc5745-mediated protective role against DOX-induced cardiomyocyte apoptosis

**Fig. 6:**
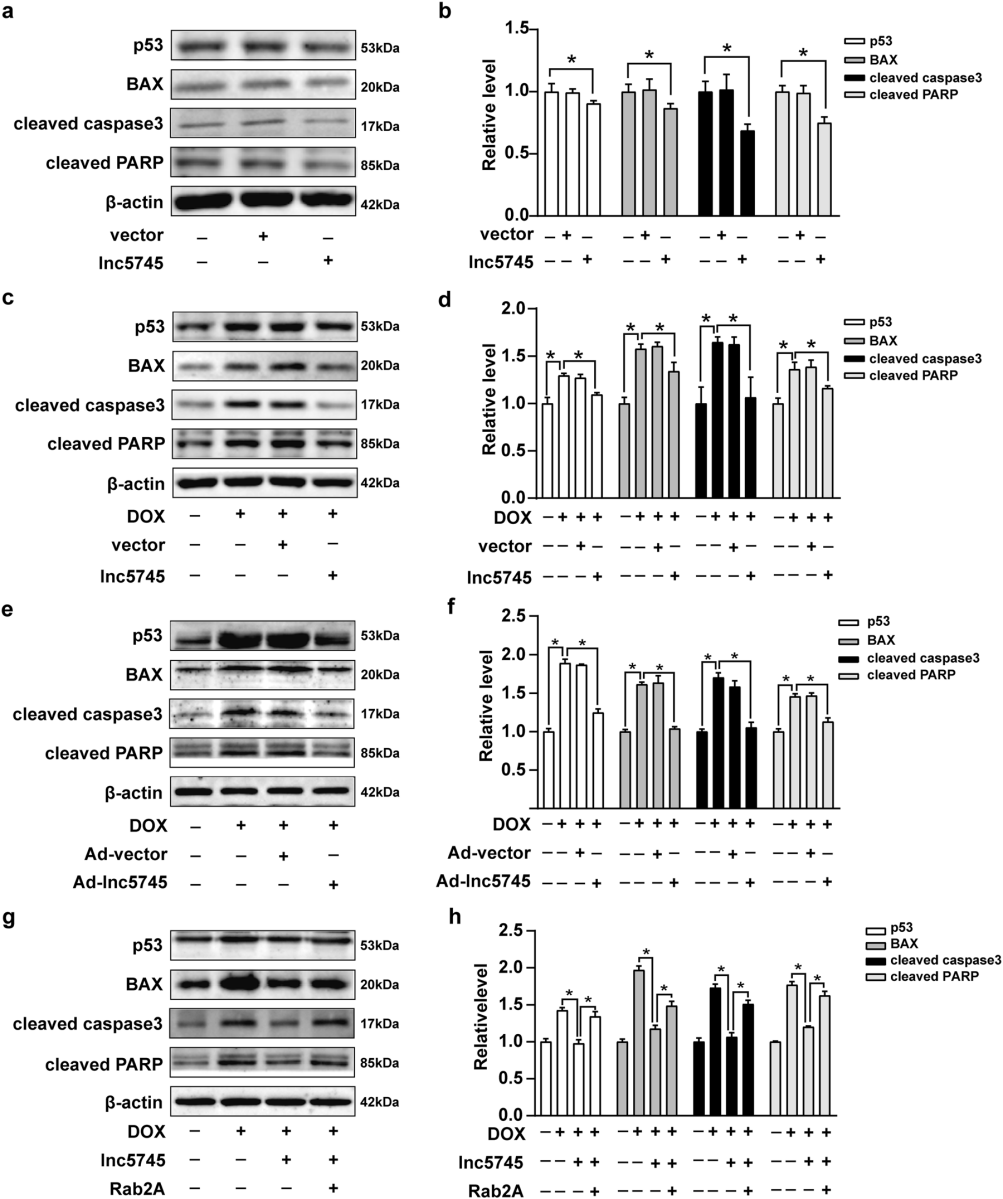
Lnc5745 regulates p53-mediated cardiomyocyte apoptosis. **a**, **b** The expression levels of p53-related apoptosis pathway proteins in NMVCs transfected with the pCDNA3.1-lnc5745 vector for 24 h. **c**, **d** Lnc5745 overexpression affected the expression levels of p53-related apoptosis pathway proteins in NMVCs treated with DOX. **e**, **f** Lnc5745 affected the levels of p53 apoptosis pathway-related proteins in mouse models. **g**, **h** Rab2A increased cardiomyocyte apoptosis attenuated by lnc5745 under DOX treatment by activating the p53/BAX/c-caspase3/c-PARP pathway. The results are from three independent experiments, *P < 0.05. Variables are presented as the mean ± SD.

p53, a tumour suppressor, is expressed at low intracellular levels and is even undetectable under normal physiological conditions. However, under conditions of intracellular stress, such as hypoxia, oncogene activation, and DNA damage, the expression of p53 might increase through posttranslational modifications [36]. p53 is involved in the pathogenesis of cardiovascular diseases through pro-apoptosis, pro-autophagy, pro-necrosis, anti-angiogenesis, metabolism regulation and cell cycle arrest regulation [37]. Among them, the p53-related apoptosis pathway is an important mechanism. p53 is mainly involved in intrinsic pathway-induced apoptosis, which is initiated from mitochondria. p53 interacts with members of the BCL-2 family (such as BAX and BAK) and finally activates caspase and cleaves PARP, causing cell apoptosis [38]. Herein, we found that overexpression of lnc5745 inhibited the expression of p53, BAX, cleaved caspase3 (c-caspase3) and cleaved PARP (c-PARP) in NMVCs (Fig. 6a, b). Importantly, overexpression of lnc5745 attenuated DOX-induced elevated expression of p53, BAX, c-caspase3 and c-PARP (Fig. 6c, d). In addition, in a mouse model, the western blot results revealed that the p53-related apoptotic pathway could be activated under DOX treatment, while overexpression of lnc5745 attenuated DOX-induced cardiomyopathy in vivo by suppressing p53-related signals, thus subsequently protecting cardiac function (Fig. 6e, f). These data indicated that the p53 apoptotic signalling pathway is responsible for the protective function of lnc5745 in DOX-induced cardiomyocyte apoptosis. Lnc5745 attenuated DOX-induced cardiomyocyte apoptosis, while Rab2A could increase apoptosis by activating the p53 pathway, reversing the protective effect of lnc5745 (Fig. 6g, h). Overall, the p53 apoptotic signalling pathway was responsible for the lnc5745-mediated protective role in DOX-induced cardiomyocyte apoptosis, and Rab2A antagonized the effect of lnc5745.

### Rab2A promotes phosphorylation of p53 on Ser 33

**Fig. 7:**
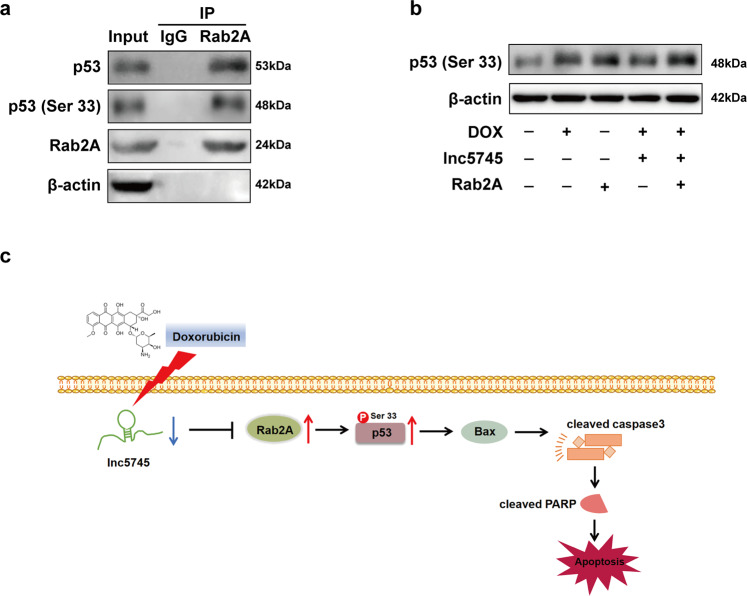
Rab2A promotes phosphorylation of p53 on Ser 33. **a** Rab2A specifically interacts with p53 and p53 (Phospho-Ser 33). **b** Effects of DOX, lnc5745 and Rab2A on p53 (Phospho-Ser 33) in NMVCs. The results are from three independent experiments, *P < 0.05. Variables are presented as the mean ± SD.

Further, we deeply studied the molecular mechanism of how Rab2A activates the p53 signalling pathway. The regulation of p53-mediated cell signal transduction pathways by biological macromolecules is very complex. Most of the existing studies have focused on the regulation of the post-translational modifications of p53 such as phosphorylation, acetylation, and ubiquitination. Phosphorylation was the first discovered post-translational modification of p53. Under stress conditions, p53 is phosphorylated on multiple sites by various kinases. Phosphorylation on Ser 33, Ser 37, Ser 46 and Thr 81 stabilizes p53 and promotes p53 transcriptional activity, so as to regulate p53-mediated cell-cycle arrest and apoptosis. Phosphorylation on Ser 351 activates p53-mediated transcriptional regulation of cellular apoptosis and cell cycle [39]. In this study, we found that Rab2A specifically binds to p53 and p53 (Phospho-Ser 33) in NMVCs (Fig. 7a). The expression of p53 (Phospho-Ser 33) was increased in DOX-treated primary cardiomyocytes. Moreover, lnc5745 overexpression can alleviate the increase of p53 (Phospho-Ser 33). However, overexpression of Rab2A increases the expression of p53 (Phospho-Ser 33) and invalidates the protective effect of lnc5745 (Fig. 7b). These data suggest that Rab2A interacts with p53 and p53 (Phospho-Ser 33), thereby activating the p53-related apoptotic pathway.

Taken together, knockdown of lnc5745 leads to increased Rab2A expression in DOX treated cardiomyocytes. Rab2A interacts with p53 and p53 (Phospho-Ser 33) to promote p53 activation, thus activating the apoptotic pathway to mediate cardiomyocytes apoptosis (Fig. 7c).

## Discussion

DOX is currently the first-line drug for the clinical treatment of cancer. However, DOX-induced cardiotoxicity is a major challenge and it severely limits its clinical application. The risk of dilated DCM increases with both the Dox treatment concentration and duration, and delayed onset of DCM can often occur years after the initial treatment [40]. It is urgent to discover the underlying molecular mechanisms of DOX-induced cardiotoxicity to attenuate cardiotoxicity and facilitate the clinical efficacy of DOX.

LncRNAs are a class of RNA molecules with limited protein-coding potential. Increasing evidence shows that lncRNAs play crucial roles in the pathophysiological processes of cardiovascular disease. LncRNA GAS5 (growth arrest-specific 5) reduces MI‐induced cardiomyocyte apoptosis by downregulating semaphorin3a (Sema3a) [41]. The lncRNA KCNQ1OT1 directly targets the FUS protein and negatively regulates its protein level, thus facilitating cardiomyocyte apoptosis in heart failure [42]. LncRNA H19 alleviates cardiomyocyte apoptosis and myocardial I/RI by inhibiting the miR-877-3p-Bcl-2-mediated mitochondrial apoptotic pathway [43]. In this study, we found that lnc5745 significantly protected against DOX-induced cardiotoxicity by alleviating cellular apoptosis in cardiomyocytes.

Existing evidence suggests that DOX-induced cardiotoxicity is related to endoplasmic reticulum stress, DNA damage, mitochondrial dysfunction and cell apoptosis [44, 45]. Apoptosis is the most important way for DOX to cause cardiomyocyte death and heart disorders [46]. In the present study, we characterized lnc5745 as a negative regulator of cardiomyocyte apoptosis. Lnc5745 is a lncRNA with a coding gene located on mouse chromosome 12 and a total length of approximately 1292 nucleotides. Thus far, there is no basic functional research report about lnc5745. Our studies demonstrated that the expression of lnc5745 was downregulated in DOX-treated NMVCs and a mouse model. Overexpression of lnc5745 alleviated cardiomyocyte apoptosis in DOX-induced cardiotoxicity in NMVCs, while knockdown of lnc5745 exacerbated the cardiotoxicity of DOX. Consistent with the above in vitro data, overexpression of lnc5745 exerted similar beneficial effects on DOX-induced cardiomyocyte apoptosis in mice. Mechanistically, we identified that lnc5745 activates the p53-related signalling pathway by binding to the Rab2A protein to alleviate DOX-induced cardiomyocyte apoptosis.

LncRNAs can bind to specific proteins to exert various kinds of functions according to their subcellular location. For instance, lncRNAs distributed in the nucleus can function as players to regulate epigenetic regulators or transcription factors, as well as to modulate chromosomal instability [47, 48], while cytoplasmic lncRNAs may participate in regulating protein stability and modifications [49, 50]. In accordance with our recent study, we found that lnc5745 was located predominantly in the cytoplasm. Moreover, RNA pulldown and MS analyses in NMVCs identified Rab2A as a protein that combined with lnc5745. Previous studies have demonstrated that lncRNAs may participate in the stabilization and degradation of targeted proteins, thereby regulating their expression level and functions [51, 52]. We found that lnc5745 functions as an upstream regulator of Rab2A and negatively regulates Rab2A expression. Nevertheless, the mechanism by which lnc5745 regulates Rab2A expression and function needs to be further examined.

Rab2A is a member of the Rab family, known alternatively as a small guanosine triphosphate (GTP)-binding protein and is reported to be involved in vesicle transport and autophagosome and autolysosome formation [53, 54]. More than 60 Rab isoforms have been discovered in the Golgi, which may independently regulate the dense morphology of the Golgi in mammalian cells by interacting with a single isoform-specific Rab effector molecule. However, the role of Rab proteins in heart disease is rarely reported. Rab1A overexpression increases Golgi stacks in ventricular myocytes, increases transitional vesicles, and degenerates myelin sheath morphology in atrial myocytes, with abnormal subcellular distribution of PKC, which ultimately leads to cardiac hypertrophy and even heart failure [55]. Rab2A is reported to participate in the occurrence and invasion of a variety of cancers, especially breast cancer [56, 57]. We found that Rab2A knockdown reduced DOX-induced cardiomyocyte apoptosis and that Rab2A overexpression attenuated the protective effect of lnc5745 overexpression on DOX-induced cardiomyocyte apoptosis. Rab2A binds to p53 and p53 (Phospho-Ser 33), activates the p53-related signalling pathway, thereby promoting cardiomyocyte apoptosis. The functions of Rab2A in the heart need further research.

Previous studies have shown that DOX directly or indirectly causes DNA double-strand breaks by interfering with the action of topoisomerase II-α and oxidative stress induced by Rac1 [58]. DNA damage can activate ataxia telangiectasia mutated kinase (ATM) and extracellular signal-regulated kinase 1/2 (ERK1/2) [59], increase the activity of p53, further stimulate the translocation of BAX/BAK from the cytoplasm to the outer mitochondrial membrane, and cause the release of cytochrome C and the activation of the apoptotic executive protein caspase3/7, as well as the cleavage of PARP, eventually leading to cell apoptosis. In our study, we found that overexpression of lnc5745 downregulated the expression of some vital proteins in the p53-related apoptosis pathway in cardiomyocytes, while overexpression of Rab2A attenuated the regulatory effect of lnc5745. We thus infer that lnc5745 regulates the p53-related apoptotic pathway by targeting Rab2A, thereby playing a protective role against DOX-induced cardiomyocyte apoptosis.

In conclusion, we identified lnc5745 as a novel lncRNA related to DOX-induced myocardial apoptosis. Lnc5745 negatively regulates Rab2A and further inhibits the activation of the p53-related apoptotic pathway, thereby protecting cardiomyocytes from apoptosis. Our studies provide new insight for investigating the function and molecular mechanism of lncRNAs in DOX-induced cardiomyocyte apoptosis. The development of novel therapeutic strategies targeting the lnc5745-Rab2A-p53 axis might provide new insight into DOX-induced cardiotoxicity in cancer treatment.

## Materials and methods

### Cardiomyocytes culture and treatment

Cardiomyocytes were isolated from 1- to 2-day neonatal mice. First, the thoraxes of the mouse pups were opened, and the hearts were surgically removed. After cleaning away the connective tissue and blood clots, the hearts were minced in phosphate buffer. Heart pieces were digested at 37 °C in HEPES-buffered saline solution containing 1.2 mg/mL pancreatin and 0.14 mg/ml collagenase (Worthington, USA) repetitively. Subsequently, the mixture was collected and centrifuged at 1000 rpm for 5 min. Next, the cells were resuspended in Dulbecco’s modified Eagle’s medium/F-12 (Gibco, USA) containing 5% heat-inactivated foetal bovine serum, 100 U/mL penicillin, 100 μg/mL streptomycin, and 0.1 mM bromodeoxyuridine. The dissociated cells were placed at 37 °C for 1.5 h to separate the cardiac fibroblasts from the cardiomyocytes. The cells were then diluted to 1 × 10^6^ cells/ml in 10 cm culture dishes according to the specific experimental requirements. In this study, the cells were treated with 1 μM DOX for 24 h before being used for detection in the DOX group. Primary cardiomyocytes were identified by immunofluorescence. All procedures were in agreement with the standards for care of laboratory animals as outlined in the NIH Guide for the Care and Use of Laboratory Animals. All animal experiments were approved by the Animal Ethics Committee of Qingdao University and performed in accordance with the Administration of Affairs Concerning Experimental Animals of China.

### Microarray analysis

The Affymetrix Mouse Clariom™ D Assay was used in the experiment and data analysis. Total RNAs was quantified by the NanoDrop ND-2000 (Thermo Scientific) and the RNAs integrity was assessed using Agilent Bioanalyzer 2100 (Agilent Technologies). The sample labelling, microarray hybridization and washing were performed based on the manufacturer’s standard protocols. Briefly, total RNAs were transcribed to double strand cDNAs and then synthesized cRNAs. Next, 2nd cycle cDNAs were synthesized from cRNAs. Followed fragmentation and biotin labelling, the 2nd cycle cDNAs were hybridized onto the microarray. After washing and staining, the arrays were scanned by the Affymetrix Scanner 3000 (Affymetrix).

Affymetrix GeneChip Command Console (version 4.0, Affymetrix) software was used to extract raw data. Next, Expression Console (version1.3.1, Affymetrix) software offered RMA normalization for both gene and exon level analysis. Genesrping software (version 13.1, Agilent Technologies) was employed to finish the gene expression analysis. Differentially expressed genes were then identified through fold change as well as *P* value calculated with *t*-test. The threshold set for up- and down-regulated genes was a fold change > = 2.5 and a *P* value < = 0.05. Afterwards, GO analysis and KEGG analysis were applied to determine the roles of these differentially expressed mRNAs played in these GO terms or pathways. Finally, Hierarchical Clustering was performed to display the distinguishable genes’ expression pattern among samples.

### Quantitative reverse transcription-PCR

Total RNA was extracted using TRIzol reagent (Vazyme, China). RNA (1 μg) was reverse transcribed with a Prime Script RT Reagent Kit (Takara, Japan). Real-time qPCR was performed using TB GreenTM Premix Ex Taq IITM (Takara, Japan). The following primers were used for qPCR: lnc5745 (5′-GGTCACATCTCAATGCCACTC-3′ and 5′-AATTTCTCCTCTTTCCCCAGT-3′); Rab2A (5′-GCTTTTGCACGAGAGCATGG-3′ and 5′-CCATGAGATGCGTTGGTAGC-3′); and GAPDH (5′-AGGTCGGTGTGAACGGATTTG-3′ and 5′-TGTAGACCATGTAGTTGAGGTCA-3′).

### Fluorescence in situ hybridization

The RNA fluorescence in situ hybridization (FISH) assay was performed using a Fluorescent In Situ Hybridization Kit (GenePharma, China). Cy5-labelled lnc5745 probe (Sangon, China) sequences were designed as follows: 5′ Cy5-CAAGTGCCCAATTTCTCCTCTTTCCCCAGT-3′. The cells were observed and imaged under a laser confocal fluorescence microscope.

### Overexpression and RNA interference

For the construction of the lnc5745-overexpressing vector, full-length lnc5745 was synthesized and inserted into the *Xba* I restriction site of the pCDNA3.1 plasmid using an EasyGeno Assembly Cloning Kit (Tiangen, China). Empty pCDNA3.1 plasmids were used as a negative control. The Rab2A overexpression vector was constructed using the same strategy. Lnc5745 siRNA (si-5745) and negative control siRNA (si-NC) were designed and synthesized by GenePharma (China). The sequences of si-5745 were GCUCUUAUUUCCUCCUCUATT (sequence 1) and UAGAGGAGGAAAUAAGAGCTT (sequence 2). The sequences of si-NC were UUCUUCGAACGUGUCACGUTT (sequence 1) and ACGUGACACGUUCGGAGAATT (sequence 2). Lipofectamine 3000 Transfection Reagent (Invitrogen, USA) was used for the transfection of plasmids and siRNAs into cells following the manufacturer’s protocol. Experiments were performed 24 hours after transfection.

### Cell Counting Kit-8

Cell viability was assessed using Meilun Cell Counting Kit-8 (Meilun Bio, China). We plated 5000 cardiomyocytes per well in a 96-well tissue culture microplate. Cardiomyocytes were treated or transfected under the indicated conditions. Then, 10 μL WST-8 solution was added to each well of the plate. The cardiomyocytes were protected from light and incubated with WST-8 for 2 h at 37 °C. The absorbance was measured at 450 nm using a microplate reader.

### Apoptosis assay

Caspase 3/7 activity was detected using a Caspase 3/7 Activity Assay Kit (Meilun Bio, China) according to the manufacturer’s instructions. TUNEL staining was performed using the TUNEL Apoptosis Detection Kit (YEASEN, China) according to the manufacturer’s instructions. Samples were mounted with mounting medium containing 4’,6’-diamidino-2-phenylindole (DAPI) and observed under a fluorescence microscope. TUNEL-positive cells were counted under the microscope by researchers who were blinded to the treatment allocation of this study. The rate of apoptotic cell nuclei was calculated by dividing the total number of TUNEL-positive nuclei by the total number of DAPI-stained nuclei.

### Western blot analysis

Total protein samples were extracted from NMVCs and mouse cardiac tissues with RIPA lysis buffer (Solarbio, China) and quantified using a bicinchoninic acid (BCA) protein quantification kit (Vazyme, China). Total protein (20-30 μg) was separated on a 12% SDS-polyacrylamide gel (SDS-PAGE) and subsequently transferred to PVDF membranes (Roche, UK). After blocking for 2 h at room temperature, the membranes were incubated overnight at 4 °C with primary antibodies against the following proteins: p53 (Cat#P100051, 1:1000, KleanAB, China), BAX (Cat#P100045, 1:1000, KleanAB, China), cleaved-caspase3 (#14220, 1:1000, CST, USA), cleaved-PARP (Cat#WL01932, 1:1000, Wanlei, China), β-actin (Cat#AC026, 1:50000, ABclonal, China), Rab2A (Cat#P101097, 1:1000, KleanAB, China), and p53 (Phospho-Ser 33) (YP0208, 1:1000, Immunoway, China). The membranes were then exposed to an HRP-conjugated secondary antibody for 1 h. The ECL chemiluminescence method was used for western blot analysis.

### RNA pulldown

Biotinylated lnc5745 or biotinylated negative control probes were designed and synthesized by Sangon Biotech. The sequence of the biotinylated lncRNA 5745 probe was: 5′-AGGGTGTTTAGAAAGCTCCG-3′. The sequence of the biotinylated negative control probe was: 5′-TTGAGTGCGAAGATCACGGT-3′. All procedures were performed under RNase-free conditions. The NMVCs were lysed in RIPA lysis buffer (Solarbio, China) and centrifuged at 12,000 *g* for 20 min at 4 °C. The protein-containing supernatants were collected. Then, the protein-containing supernatants were incubated with the biotinylated probes for 12 h at 4 °C. Streptavidin magnetic beads were added to each binding reaction and incubated for 4 h at 4 °C. The beads were washed five times with high-salt buffer and then twice with low-salt buffer. The samples were then boiled for 10 min and resolved via SDS–PAGE.

### Mass spectrometry

The SDS–PAGE gel was stained with Coomassie Brilliant Blue. The entire strip of samples was excised from the gel and first subjected to enzymatic hydrolysis with trypsin for 20 h at 37 °C. Then, liquid chromatography coupled with tandem mass spectrometry (LC–MS/MS) was used to analyse the enzymatically digested samples. Finally, mass spectrometry matching software such as MASCOT was used to analyse the LC–MS/MS data to obtain qualitative identification information of the target protein and peptide molecules. The specific analysis process was completed by Shanghai Applied Protein Technology (APT), China.

### RNA immunoprecipitation

All procedures were performed under RNase-free conditions. The NMVCs were lysed in RIPA lysis buffer (Solarbio, China) for 30 min and centrifuged at 12,000 *g* for 20 min at 4 °C. Protein-containing supernatants were separated from the precipitate and collected. The cardiomyocyte lysates were incubated with 8 μL anti-Rab2A antibody (Cat#P101097, KleanAB, China) or IgG antibody (Cat#AS003, ABclonal, China) overnight at 4 °C. Agarose beads were added to each sample and incubated for 4 h at 4 °C. After washing off any unbound materials, RNAs binding to Rab2A were extracted with a standard TRIzol (Vazyme, China) RNA extraction protocol and examined by qRT–PCR and PCR.

### Animal experiments

Eight-week-old male C57BL/6 J mice were divided into four groups, and the grouping was randomized and double-blind, with 6 mice in each group. Prior to experiments, the mice were raised in a specific pathogen free environment with comfortable temperature, sterile feed, and drinking water, and an alternating 12 h day–night cycle for 7 days. Mice received a single intravenous injection of saline, adenovirus empty vector (Ad-vector) or adenovirus lnc5745 (Ad-lnc5745) vector (Hanheng, China) with approximately 1 × 10^11^ plaque-forming units (pfu) via the tail vein. Four days later, the mice received a single intraperitoneal injection of saline or DOX (20 mg/kg). After 7 days, the mice were anaesthetized with 1.5% isoflurane, and echocardiography was performed to examine cardiac function indices, including the ejection fraction of the left ventricular diameter (EF), fractional shortening of the left ventricular diameter (FS), the systolic left ventricular internal diameter (LVIDs) and the diastolic left ventricular internal diameters (LVIDd). The mice were then sacrificed, and their hearts were quickly isolated, fixed in 4% paraformaldehyde or stored at −80 °C for subsequent experiments. Apoptosis in the myocardial tissues was measured using the TUNEL method with an in situ apoptosis detection kit (YEASEN, China). To evaluate the extent of cardiac fibrosis, Masson trichrome staining was performed using a staining kit (Solarbio, China) following the manufacturer’s instructions. Cardiac fibrosis was stained blue with Masson’s trichrome. The percentage of fibrotic tissue was calculated as the collagen area (blue) divided by the total cardiac area on Masson’s trichrome-stained sections.

### Statistical analysis

The sample size was chosen according to previous observations, which perform similar experiments to see significant results, or the results from our preliminary experiments. Statistical analyses were performed using GraphPad Prism (Version 8, GraphPad Software), and the results are presented as the means ± standard deviation, *n* = 3. Each experiment was repeated at least three times. Data with normal distribution and homogeneity of variance between two groups were evaluated utilizing unpaired *t*-test. Data comparisons among multiple groups were analyzed using one-way analysis of variance (ANOVA) or repeated-measures ANOVA, followed by Tukey’s post hoc test. *P* < 0.05 was defined as statistically significant.

## Supplementary information

co-authors' email responses

Original Data File

Supplementary Figure Legends

Supplementary Figure S1 The effect of lnc5745 overexpression and knockdown on cardiomyocyte apoptosis.

Supplementary Figure S2 Effect of NH_4_Cl on the stability of Rab2A protein.

## Data Availability

All data and materials are available under request by contacting with the corresponding author.

## References

[1] Volkova M, Russell R (2011). Anthracycline cardiotoxicity: prevalence, pathogenesis and treatment. Curr Cardiol Rev.

[2] Ferrans VJ, Clark JR, Zhang J, Yu ZX, Herman EH (1997). Pathogenesis and prevention of doxorubicin cardiomyopathy. Tsitologiia..

[3] Carvalho C, Santos RX, Cardoso S, Correia S, Oliveira PJ, Santos MS (2009). Doxorubicin: the good, the bad and the ugly effect. Curr Med Chem.

[4] Wang Y, Lei T, Yuan J, Wu Y, Shen X, Gao J (2018). GCN2 deficiency ameliorates doxorubicin-induced cardiotoxicity by decreasing cardiomyocyte apoptosis and myocardial oxidative stress. Redox Biol.

[5] Zhang Y, Shan C, Wang Y, Qian L, Jia D, Zhang Y (2020). Cardiovascular toxicity and mechanism of bisphenol A and emerging risk of bisphenol S. Sci Total Environ.

[6] McGowan JV, Chung R, Maulik A, Piotrowska I, Walker JM, Yellon DM (2017). Anthracycline Chemotherapy and Cardiotoxicity. Cardiovasc Drugs Ther.

[7] Singal PK, Deally CM, Weinberg LE (1987). Subcellular effects of adriamycin in the heart: a concise review. J Mol Cell Cardiol.

[8] Nithipongvanitch R, Ittarat W, Cole MP, Tangpong J, Clair DK, Oberley TD (2007). Mitochondrial and nuclear p53 localization in cardiomyocytes: redox modulation by doxorubicin (Adriamycin)?. Antioxid Redox Signal.

[9] Sterba M, Popelova O, Vavrova A, Jirkovsky E, Kovarikova P, Gersl V (2013). Oxidative stress, redox signaling, and metal chelation in anthracycline cardiotoxicity and pharmacological cardioprotection. Antioxid Redox Signal.

[10] Octavia Y, Tocchetti C, Gabrielson K, Janssens S, Crijns H, Moens AL (2012). Doxorubicin-induced cardiomyopathy: from molecular mechanisms to therapeutic strategies. J Mol Cell Cardiol.

[11] Wan Q, Xu T, Ding W, Zhang X, Ji X, Yu T (2018). miR-499-5p Attenuates Mitochondrial Fission and Cell Apoptosis via p21 in Doxorubicin Cardiotoxicity. Front Genet.

[12] Cardon T, Franck J, Coyaud E, Laurent EMN, Damato M, Maffia M (2020). Alternative proteins are functional regulators in cell reprogramming by PKA activation. Nucleic Acids Res.

[13] Guo FX, Wu Q, Li P, Zheng L, Ye S, Dai XY (2019). The role of the LncRNA-FA2H-2-MLKL pathway in atherosclerosis by regulation of autophagy flux and inflammation through mTOR-dependent signaling. Cell Death Differ.

[14] Van Grembergen O, Bizet M, de Bony EJ, Calonne E, Putmans P, Brohee S (2016). Portraying breast cancers with long noncoding RNAs. Sci Adv.

[15] Liu CY, Zhang YH, Li RB, Zhou LY, An T, Zhang RC (2018). LncRNA CAIF inhibits autophagy and attenuates myocardial infarction by blocking p53-mediated myocardin transcription. Nat Commun.

[16] Klattenhoff CA, Scheuermann JC, Surface LE, Bradley RK, Fields PA, Steinhauser ML (2013). Braveheart, a long noncoding RNA required for cardiovascular lineage commitment. Cell.

[17] Kurian L, Aguirre A, Sancho-Martinez I, Benner C, Hishida T, Nguyen T (2015). Identification of novel long noncoding RNAs underlying vertebrate cardiovascular development. Circulation.

[18] Nelson B, Makarewich C, Anderson D, Winders B, Troupes C, Wu F (2016). A peptide encoded by a transcript annotated as long noncoding RNA enhances SERCA activity in muscle. Science.

[19] Zhang Y, Jiao L, Sun L, Li Y, Gao Y, Xu C (2018). LncRNA ZFAS1 as a SERCA2a Inhibitor to Cause Intracellular Ca(2+) Overload and Contractile Dysfunction in a Mouse Model of Myocardial Infarction. Circ Res.

[20] Wang K, Liu F, Liu CY, An T, Zhang J, Zhou LY (2016). The long noncoding RNA NRF regulates programmed necrosis and myocardial injury during ischemia and reperfusion by targeting miR-873. Cell Death Differ.

[21] Chao CC (2015). Mechanisms of p53 degradation. Clin Chim Acta.

[22] Castrogiovanni C, Waterschoot B, De Backer O, Dumont P (2018). Serine 392 phosphorylation modulates p53 mitochondrial translocation and transcription-independent apoptosis. Cell Death Differ.

[23] Tomita Y, Marchenko N, Erster S, Nemajerova A, Dehner A, Klein C (2006). WT p53, but not tumor-derived mutants, bind to Bcl2 via the DNA binding domain and induce mitochondrial permeabilization. J Biol Chem.

[24] Xu H, Tai J, Ye H, Kang CB, Yoon HS (2006). The N-terminal domain of tumor suppressor p53 is involved in the molecular interaction with the anti-apoptotic protein Bcl-Xl. Biochem Biophys Res Commun.

[25] Pietsch EC, Perchiniak E, Canutescu AA, Wang G, Dunbrack RL, Murphy ME (2008). Oligomerization of BAK by p53 utilizes conserved residues of the p53 DNA binding domain. J Biol Chem.

[26] Carneiro BA, El-Deiry WS (2020). Targeting apoptosis in cancer therapy. Nat Rev Clin Oncol.

[27] Kale J, Osterlund EJ, Andrews DW (2018). BCL-2 family proteins: changing partners in the dance towards death. Cell Death Differ.

[28] Zhu W, Soonpaa M, Chen H, Shen W, Payne R, Liechty E (2009). Acute doxorubicin cardiotoxicity is associated with p53-induced inhibition of the mammalian target of rapamycin pathway. Circulation.

[29] Shizukuda Y, Matoba S, Mian O, Nguyen T, Hwang PJM, biochemistry c. Targeted disruption of p53 attenuates doxorubicin-induced cardiac toxicity in mice. Mol Cell Biochem. 2005;273:25–32. 10.1007/s11010-005-5905-8 16013437

[30] Sardão V, Oliveira P, Holy J, Oliveira C, Wallace KB, pharmacology. Doxorubicin-induced mitochondrial dysfunction is secondary to nuclear p53 activation in H9c2 cardiomyoblasts. Cancer Chemother Pharmacol. 2009;64:811–27. 10.1007/s00280-009-0932-x 19184017

[31] Cunha-Oliveira T, Ferreira L, Coelho A, Deus C, Oliveira PJT, pharmacology a. Doxorubicin triggers bioenergetic failure and p53 activation in mouse stem cell-derived cardiomyocytes. Toxicol Appl Pharmacol. 2018;348:1–13. 10.1016/j.taap.2018.04.009 29653124

[32] Kang YJ, Yang DC, Kong L, Hou M, Meng YQ, Wei L (2017). CPC2: a fast and accurate coding potential calculator based on sequence intrinsic features. Nucleic Acids Res.

[33] Räsänen M, Degerman J, Nissinen T, Miinalainen I, Kerkelä R, Siltanen A (2016). VEGF-B gene therapy inhibits doxorubicin-induced cardiotoxicity by endothelial protection. Proc Natl Acad Sci U S A.

[34] Sun M, Kraus WL (2015). From discovery to function: the expanding roles of long noncoding RNAs in physiology and disease. Endocr Rev.

[35] Aizawa M, Fukuda M (2015). Small GTPase Rab2B and Its Specific Binding Protein Golgi-associated Rab2B Interactor-like 4 (GARI-L4) Regulate Golgi Morphology. J Biol Chem.

[36] Meek D, Anderson CW (2009). Posttranslational modification of p53: cooperative integrators of function. Cold Spring Harb Perspect Biol.

[37] Men H, Cai H, Cheng Q, Zhou W, Wang X, Huang S (2021). The regulatory roles of p53 in cardiovascular health and disease. Cell Mol Life Sci.

[38] Bouvard V, Zaitchouk T, Vacher M, Duthu A, Canivet M, Choisy-Rossi C (2000). Tissue and cell-specific expression of the p53-target genes: bax, fas, mdm2 and waf1/p21, before and following ionising irradiation in mice. Oncogene.

[39] Kruse JP, Gu W (2008). SnapShot: p53 posttranslational modifications. Cell.

[40] Kumar S, Marfatia R, Tannenbaum S, Yang C, Avelar E (2012). Doxorubicin-induced cardiomyopathy 17 years after chemotherapy. Tex Heart Inst J.

[41] Hao S, Liu X, Sui X, Pei Y, Liang Z, Zhou N (2018). Long non-coding RNA GAS5 reduces cardiomyocyte apoptosis induced by MI through sema3a. Int J Biol Macromol.

[42] Lai L, Xu Y, Kang L, Yang J, Zhu GJE, pathology m. LncRNA KCNQ1OT1 contributes to cardiomyocyte apoptosis by targeting FUS in heart failure. Exp Mol Pathol. 2020;115:104480. 10.1016/j.yexmp.2020.104480 32497620

[43] Li X, Luo S, Zhang J, Yuan Y, Jiang W, Zhu H (2019). lncRNA H19 Alleviated Myocardial I/RI via Suppressing miR-877-3p/Bcl-2-Mediated Mitochondrial Apoptosis. Mol Ther Nucleic Acids.

[44] Varricchi G, Ameri P, Cadeddu C, Ghigo A, Madonna R, Marone G (2018). Antineoplastic Drug-Induced Cardiotoxicity: A Redox Perspective. Front Physiol.

[45] Wang P, Wang L, Lu J, Hu Y, Wang Q, Li Z (2019). SESN2 protects against doxorubicin-induced cardiomyopathy via rescuing mitophagy and improving mitochondrial function. J Mol Cell Cardiol.

[46] Kumar D, Kirshenbaum L, Li T, Danelisen I, Singal PJA, signaling r. Apoptosis in adriamycin cardiomyopathy and its modulation by probucol. Antioxid Redox Signal. 2001;3:135–45. 10.1089/152308601750100641 11291592

[47] van Heesch S, van Iterson M, Jacobi J, Boymans S, Essers P, de Bruijn E (2014). Extensive localization of long noncoding RNAs to the cytosol and mono- and polyribosomal complexes. Genome Biol.

[48] Lee JT (2012). Epigenetic regulation by long noncoding RNAs. Science.

[49] Li Z, Zhang J, Liu X, Li S, Wang Q, Chen D (2018). The LINC01138 drives malignancies via activating arginine methyltransferase 5 in hepatocellular carcinoma. Nat Commun.

[50] Jin X, Xu X, Jiang Y, Liu Y, Sun W, Guo Y (2019). The endogenous retrovirus-derived long noncoding RNA TROJAN promotes triple-negative breast cancer progression via ZMYND8 degradation. Sci Adv.

[51] Jian F, Che X, Zhang J, Liu C, Liu G, Tang Y (2021). The long-noncoding RNA SOCS2-AS1 suppresses endometrial cancer progression by regulating AURKA degradation. Cell Death Dis.

[52] Wen D, Liu WL, Lu ZW, Cao YM, Ji QH, Wei WJ (2021). SNHG9, a Papillary Thyroid Cancer Cell Exosome-Enriched lncRNA, Inhibits Cell Autophagy and Promotes Cell Apoptosis of Normal Thyroid Epithelial Cell Nthy-ori-3 Through YBOX3/P21 Pathway. Front Oncol.

[53] Ding X, Jiang X, Tian R, Zhao P, Li L, Wang X (2019). RAB2 regulates the formation of autophagosome and autolysosome in mammalian cells. Autophagy.

[54] Kajiho H, Kajiho Y, Frittoli E, Confalonieri S, Bertalot G, Viale G (2016). RAB2A controls MT1-MMP endocytic and E-cadherin polarized Golgi trafficking to promote invasive breast cancer programs. EMBO Rep.

[55] Wu G, Yussman MG, Barrett TJ, Hahn HS, Osinska H, Hilliard GM (2001). Increased myocardial Rab GTPase expression: a consequence and cause of cardiomyopathy. Circ Res.

[56] Dey K, Bharti R, Dey G, Pal I, Rajesh Y, Chavan S (2016). S100A7 has an oncogenic role in oral squamous cell carcinoma by activating p38/MAPK and RAB2A signaling pathway. Cancer Gene Ther.

[57] Luo M, Gong C, Chen C, Hu H, Huang P, Zheng M (2015). The Rab2A GTPase promotes breast cancer stem cells and tumorigenesis via Erk signaling activation. Cell Rep.

[58] Kingma P, Osheroff N. Topoisomerase II-mediated DNA cleavage and religation in the absence of base pairing. Abasic lesions a tool dissect Enzym mechanism. J Biol Chem. 1998;273:17999–8002. 10.1074/jbc.273.29.17999 9660751

[59] Liu J, Zhang J, Ren L, Wei J, Zhu Y, Duan J (2019). Fine particulate matters induce apoptosis via the ATM/P53/CDK2 and mitochondria apoptosis pathway triggered by oxidative stress in rat and GC-2spd cell. Ecotoxicol Environ Saf.

