# Supplementary material for: Long noncoding RNA NONMMUT015745 inhibits doxorubicin-mediated cardiomyocyte apoptosis by regulating Rab2A-p53 axis
Source: Cell Death Discov. 2022 Aug 16;8:364. doi: 10.1038/s41420-022-01144-9 (PMC9381503; doi:10.1038/s41420-022-01144-9)

Fig. 4 Lnc5745 targets Rab2A.

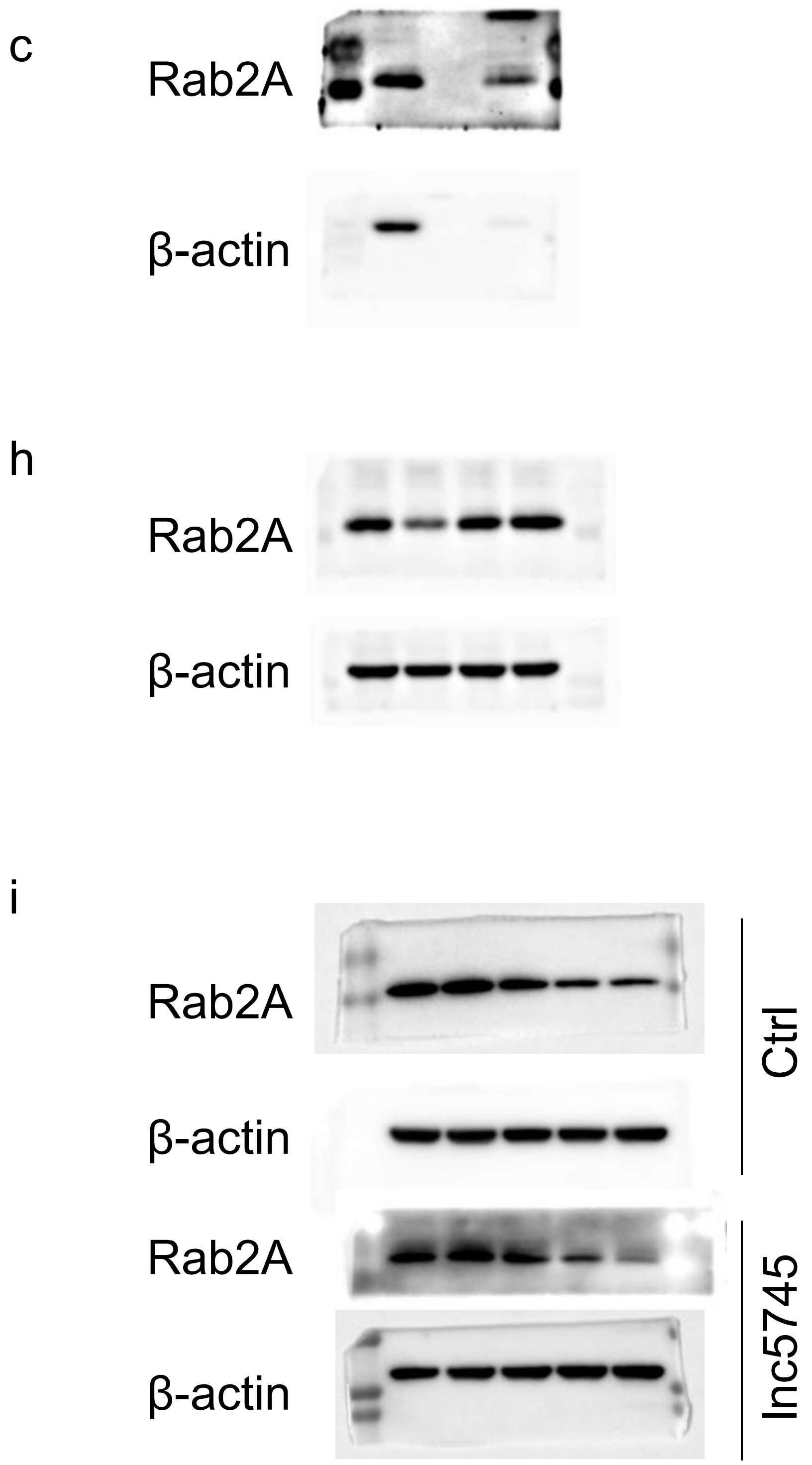

Fig. 4 Lnc5745 targets Rab2A.

j

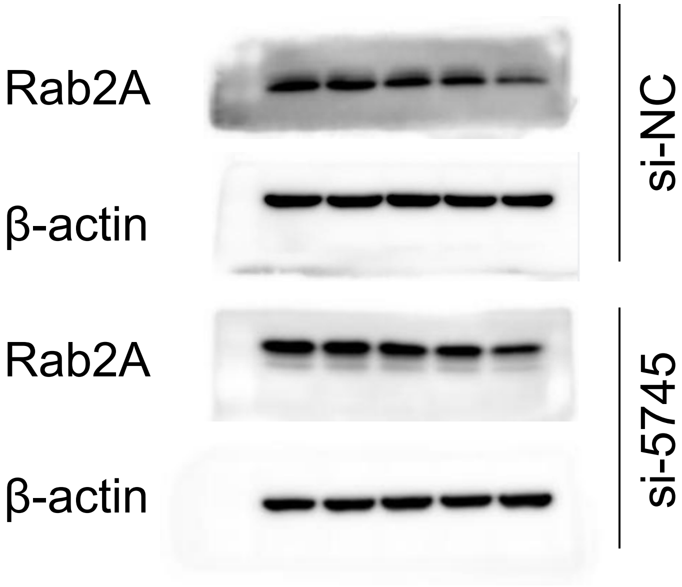

k

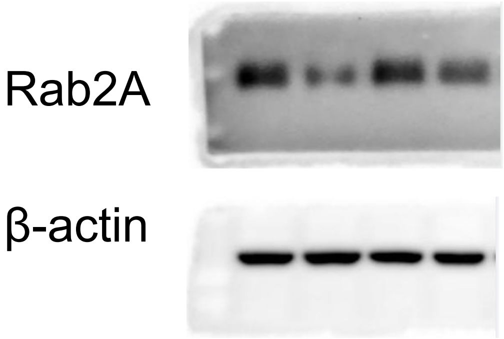

Fig. 5 Rab2A alleviates the protective effect of lnc5745 on DOX-induced apoptosis.

b

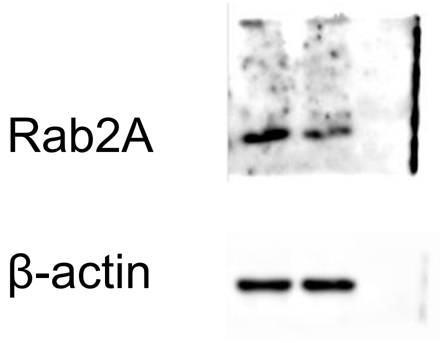

h

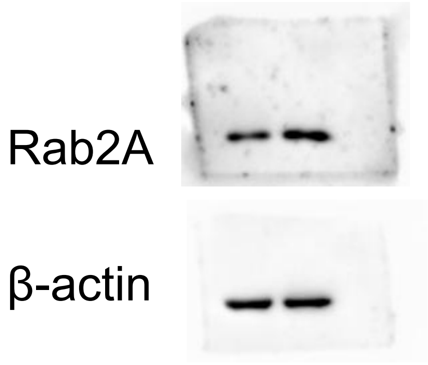

Fig. 6 Lnc5745 regulates p53-mediated cardiomyocyte apoptosis.

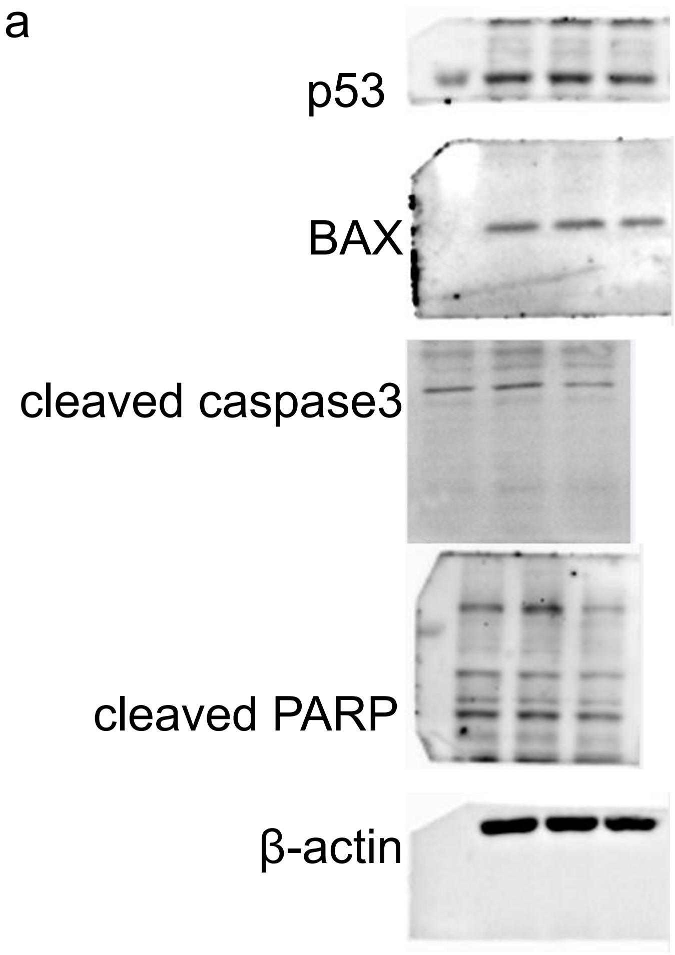

Fig. 6 Lnc5745 regulates p53-mediated cardiomyocyte apoptosis.

c

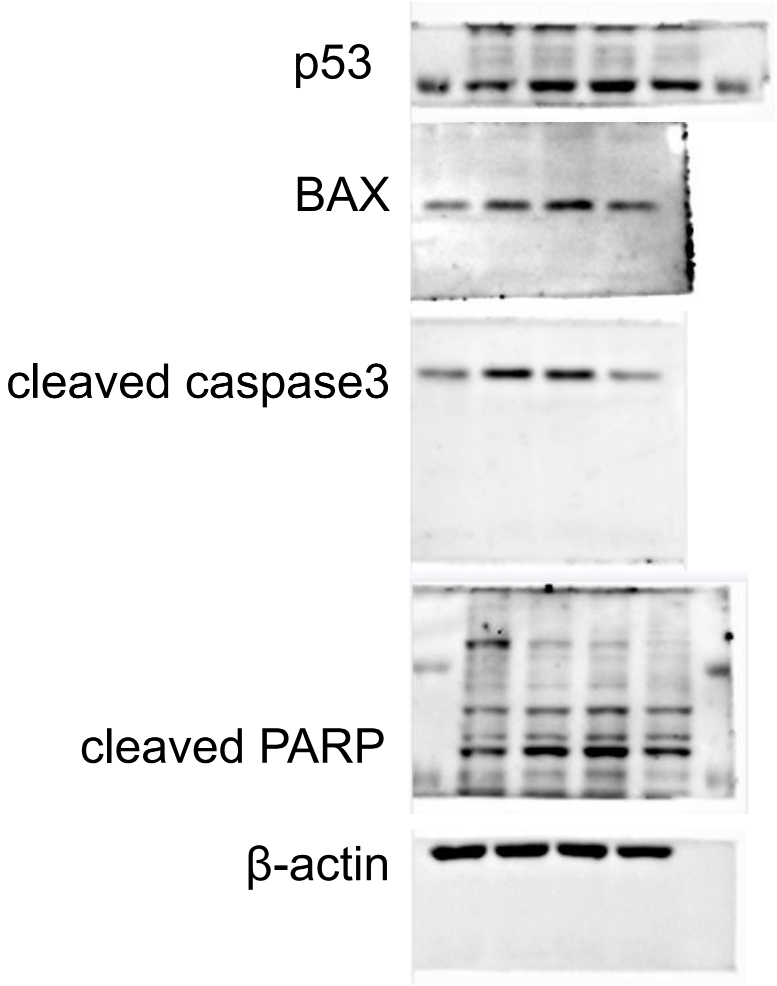

Fig. 6 Lnc5745 regulates p53-mediated cardiomyocyte apoptosis.

e

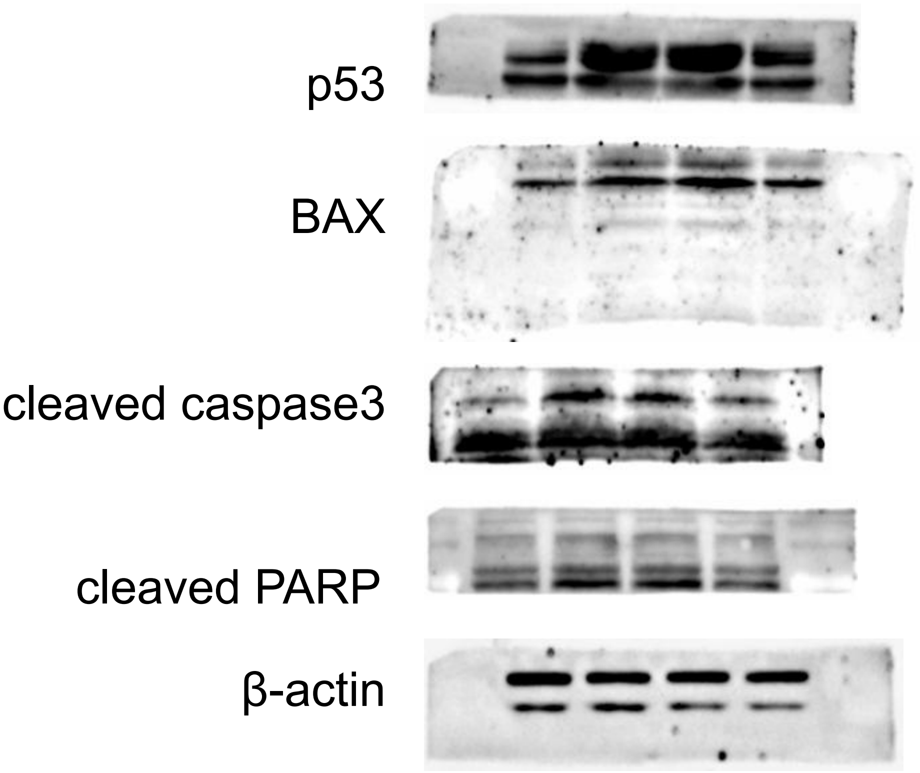

Fig. 6 Lnc5745 regulates p53-mediated cardiomyocyte apoptosis.

g

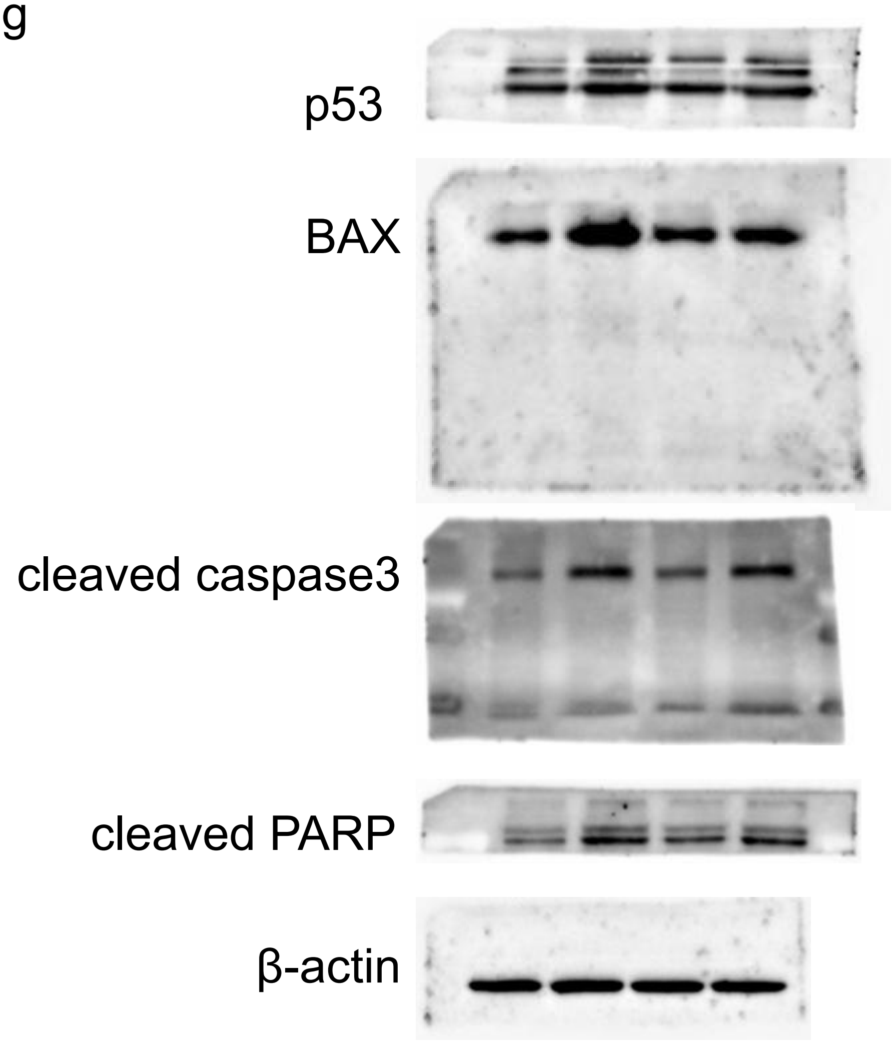

Fig. 7 Rab2A promotes phosphorylation of p53 on Ser 33.

a

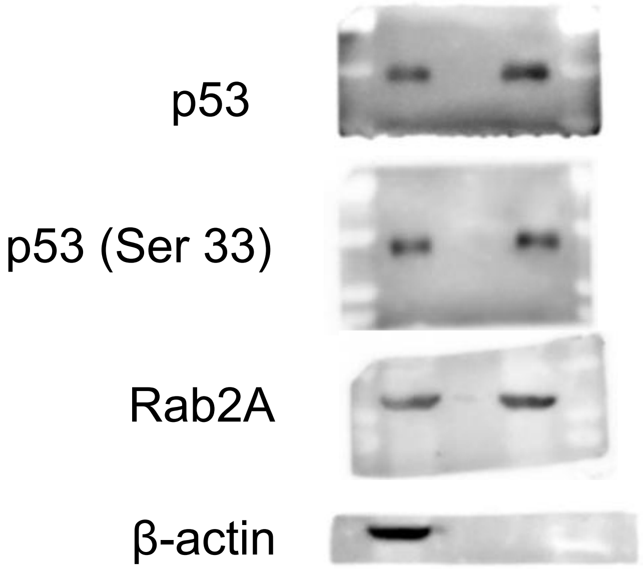

b

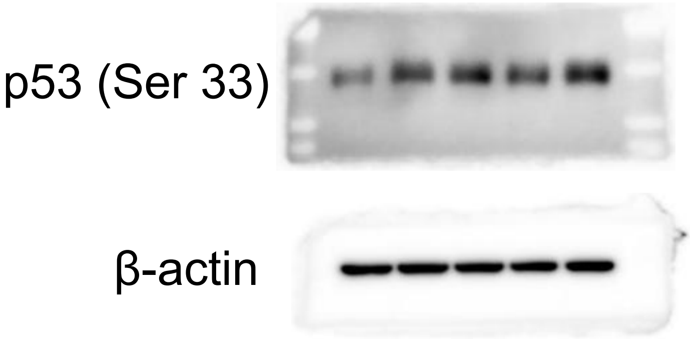

Supplementary Figure S2
Effect of NH_4Cl on the stability of Rab2A protein.

a

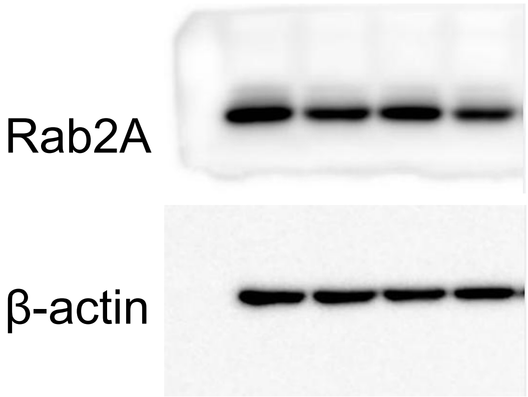

Supplement: Supplementary file 2 — Original Data File [file 41420_2022_1144_MOESM2_ESM.pdf]
