# Supplementary material for: Long noncoding RNA NONMMUT015745 inhibits doxorubicin-mediated cardiomyocyte apoptosis by regulating Rab2A-p53 axis
Source: Cell Death Discov. 2022 Aug 16;8:364. doi: 10.1038/s41420-022-01144-9 (PMC9381503; doi:10.1038/s41420-022-01144-9)
Supplement: Supplementary file 3 — Supplementary Figure Legends [file 41420_2022_1144_MOESM3_ESM.pdf]

Supplementary Figure Legends

Supplementary Figure S1 The effect of lnc5745 overexpression and knockdown on cardiomyocyte apoptosis. a RT-qPCR analysis of 10 highly downregulated lncRNAs in NMVCs treated with dox selected from lncRNA microarray data. **b** Lnc5745 was significantly upregulated in NMVCs 24h after transfection with the pCDNA3.1-lnc5745 vector. **c** CCK-8 assay showed that the overexpression of lnc5745 had no significant effect on the vitality of NMVCs. **d, e** Overexpression of lnc5745 has no obvious effect on the apoptosis of NMVCs. Scale bar, 200 μm. **f** Overexpression of lnc5745 reduced caspase3/7 activity of NMVCs. **g** Lnc5745 expression was significantly downregulated in NMVCs transfected with siRNA targeting lnc5745 (si-5745).**h** Knockdown of lnc5745 reduced the vitality of NMVCs. **i** Knockdown of lnc5745 increased caspase3/7 activity of NMVCs. **j, k** Apoptotic cells were observed with TUNEL method (green). Nucleus were stained by DAPI (blue). Lnc5745 knockdown induced apoptosis of NMVCs. Scale bar, 200 μm. The results are from three independent experiments, *P <0.05. Variables are shown as mean ± SD.

Supplementary Figure S2 Effect of NH_4Cl on the stability of Rab2A protein. a There was no significant change in the stability of Rab2A protein after NH_4Cl treatment for 12 hours. The results are from three independent experiments.
