# Supplementary figures and images for: Long noncoding RNA NONMMUT015745 inhibits doxorubicin-mediated cardiomyocyte apoptosis by regulating Rab2A-p53 axis
Source: Cell Death Discov. 2022 Aug 16;8:364. doi: 10.1038/s41420-022-01144-9 (PMC9381503; doi:10.1038/s41420-022-01144-9)

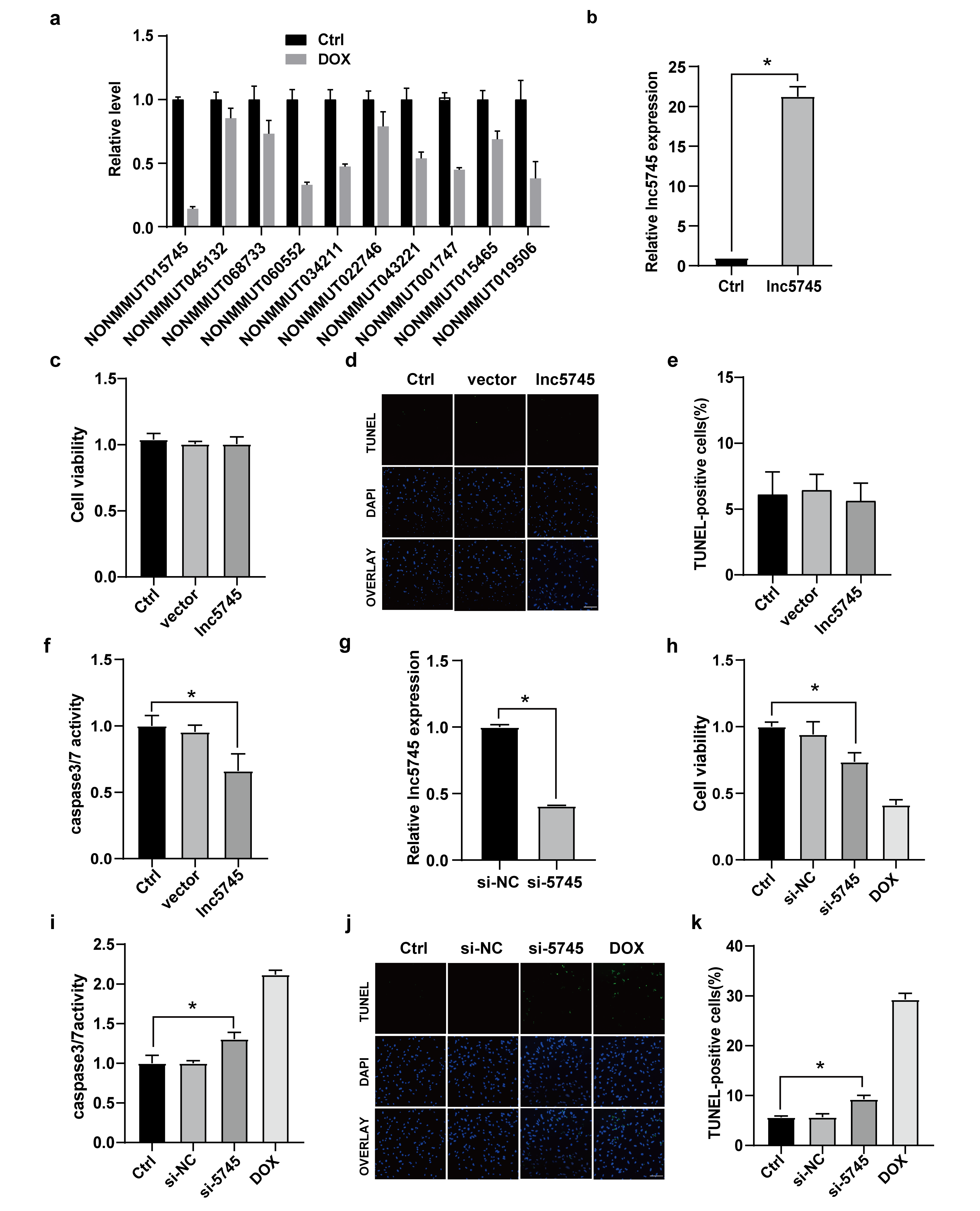

Supplement: Supplementary file 4 — Supplementary Figure S1 The effect of lnc5745 overexpression and knockdown on cardiomyocyte apoptosis. [file 41420_2022_1144_MOESM4_ESM.tif]

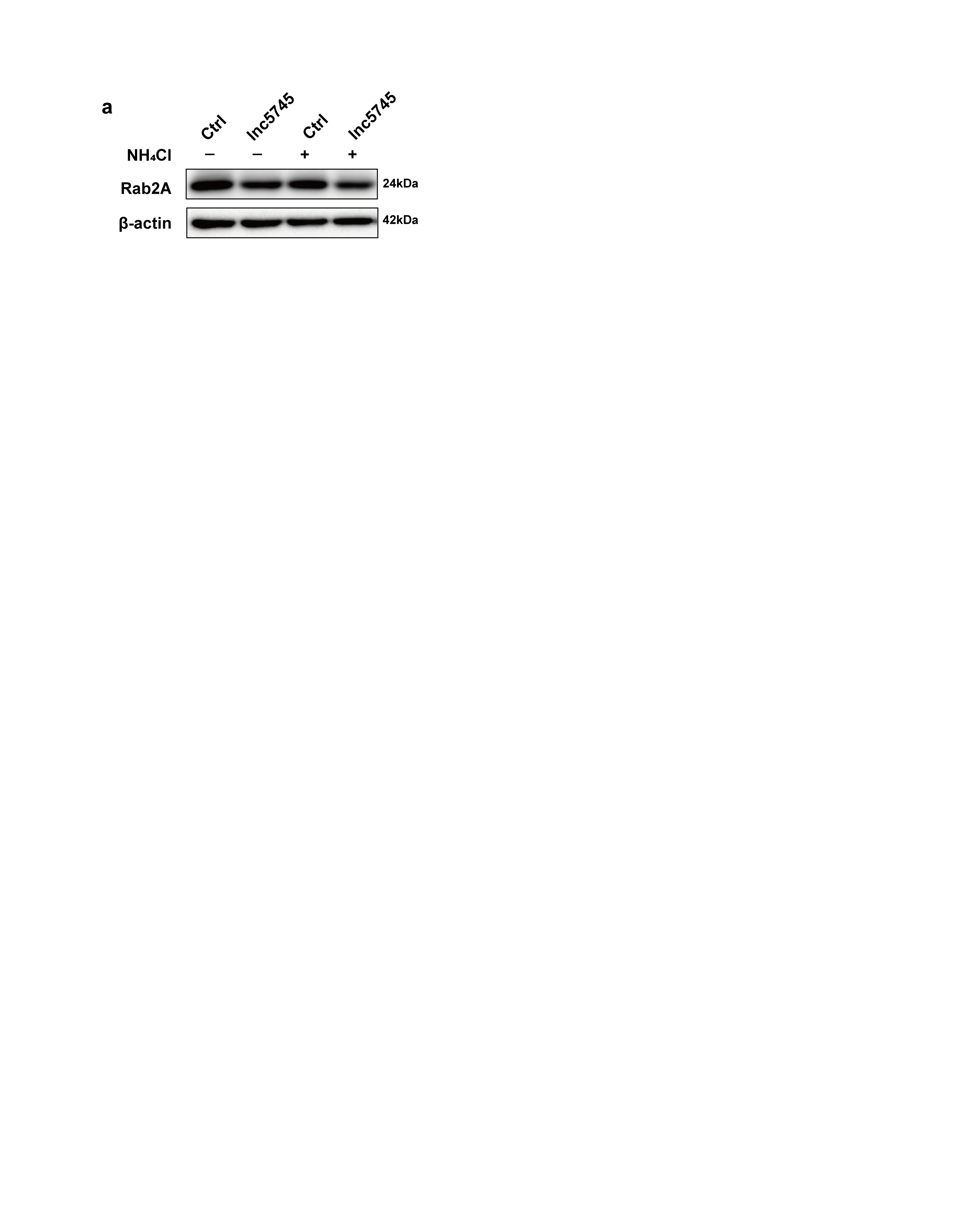

Supplement: Supplementary file 5 — Supplementary Figure S2 Effect of NH4Cl on the stability of Rab2A protein. [file 41420_2022_1144_MOESM5_ESM.tif]
