# Supplementary material for: Long noncoding RNA NONMMUT015745 inhibits doxorubicin-mediated cardiomyocyte apoptosis by regulating Rab2A-p53 axis
Source: Cell Death Discov. 2022 Aug 16;8:364. doi: 10.1038/s41420-022-01144-9 (PMC9381503; doi:10.1038/s41420-022-01144-9)
Supplement: Supplementary file 1 — co-authors' email responses [file 41420_2022_1144_MOESM1_ESM.pdf]

1. Hongjing Cai

Re:Please confirm the changes of the authors

优化[illegible]信息

发件人: Hongjing Cai<caihjing81@126.com>

收件人: 我<shanchan2019@163.com>

时　间: 2022年07月18日 13:38 (星期一)

您的邮箱安全待提升！仅需1分钟，安全性提升30%，一键升级>>

Dear Dr. Shan,

I agree to the changes of the authors.

Your sincerely,
Hongjing Cai

At 2022-07-18 13:34:58, "单婵" <shanchan2019@163.com> wrote:

- 隐藏引用文字 -

> Dear authors:
>
> The authors of our article (Long noncoding RNA NONMMUT015745 inhibits doxorubicin-mediated cardiomyocyte apoptosis by regulating Rab2A-p53 axis, CDDISCOVERY-22-3732R1) have been changed. If you confirm and agree to these changes, please reply me via email.
>
> Original authors：Hongjing Cai&,Chan Shan&*, Jie Ju,Tao Wang, Xinzhe Chen, Kai Wang, Fei Wang, Xue Yu, Shaocong Wang, Yin Wang*, Peifeng Li*（& Cai and Shan contributed equally to this work. The corresponding authors are Yin Wang, Chan Shan and Peifeng Li.)
>
> The revised authors：Hongjing Cai&, Pengchao Tian&, Jie Ju,Tao Wang, Xinzhe Chen, Kai Wang, Fei Wang, Xue Yu, Shaocong Wang, Yin Wang, Chan Shan*, Peifeng Li*（& Cai and Tian contributed equally to this work.The corresponding authors are Chan Shan and Peifeng Li.)
>
> Looking forward to receiving your reply.
>
> Yours sincerely,
> Chan Shan

2. Pengchao Tian

Re:Please confirm the changes of the authors 优化阅读 | 精简信息

发件人: Pengchao Tian<pchaotian722@yeah.net>

收件人: 我<shanchan2019@163.com>

时 间: 2022年07月18日 20:28 (星期一)

翻译成中文

您的邮箱安全待提升! 仅需1分钟，安全性提升30%，一键升级>>

Dear Dr Shan,

I confirm and agree to these changes.

Best regards,

Peng Chao Tian

At 2022-07-18 13:34:58, "单婵" <shanchan2019@163.com> wrote:

- 隐藏引用文字 -

> Dear authors:
>
> The authors of our article (Long noncoding RNA NONMMUT015745 inhibits doxorubicin-mediated cardiomyocyte apoptosis by regulating Rab2A-p53 axis, CDDISCOVERY-22-3732R1) have been changed. If you confirm and agree to these changes, please reply me via email.
>
> Original authors: Hongjing Cai&,Chan Shan&*, Jie Ju,Tao Wang, Xinzhe Chen, Kai Wang, Fei Wang, Xue Yu, Shaocong Wang, Yin Wang*, Peifeng Li* (& Cai and Shan contributed equally to this work. The corresponding authors are Yin Wang, Chan Shan and Peifeng Li.)
>
> The revised authors: Hongjing Cai&, Pengchao Tian&, Jie Ju,Tao Wang, Xinzhe Chen, Kai Wang, Fei Wang, Xue Yu, Shaocong Wang, Yin Wang, Chan Shan*, Peifeng Li* (& Cai and Tian contributed equally to this work.The corresponding authors are Chan Shan and Peifeng Li.)
>
> Looking forward to receiving your reply.
>
> Yours sincerely,
>
> Chan Shan

3. Jie Ju

Re:Please confirm the changes of the authors 优化阅读 | 精简信息

发件人：Jie Ju <jujiee@126.com>

收件人：我<shanchan2019@163.com>

时 间：2022年07月18日 19:12 (星期一)

翻译成中文

您的邮箱安全待提升！仅需1分钟，安全性提升30%，一键升级>>

Dear Dr. Shan

I agree to the changes of the authors.

Yours sincerely,

Ju Jie

At 2022-07-18 13:34:58, "单婵" <shanchan2019@163.com> wrote:

- 隐藏引用文字 -

Dear authors:

The authors of our article (Long noncoding RNA NONMMUT015745 inhibits doxorubicin-mediated cardiomyocyte apoptosis by regulating Rab2A-p53 axis, CDDISCOVERY-22-3732R1) have been changed. If you confirm and agree to these changes, please reply me via email.

Original authors：Hongjing Cai&,Chan Shan&*, Jie Ju,Tao Wang, Xinzhe Chen, Kai Wang, Fei Wang, Xue Yu, Shaocong Wang, Yin Wang*, Peifeng Li*（& Cai and Shan contributed equally to this work. The corresponding authors are Yin Wang, Chan Shan and Peifeng Li.）

The revised authors：Hongjing Cai&, Pengchao Tian&, Jie Ju,Tao Wang, Xinzhe Chen, Kai Wang, Fei Wang, Xue Yu, Shaocong Wang, Yin Wang, Chan Shan*, Peifeng Li*（& Cai and Tian contributed equally to this work.The corresponding authors are Chan Shan and Peifeng Li.）

Looking forward to receiving your reply.

Yours sincerely,

Chan Shan

4. Tao Wang

Re:Please confirm the changes of the authors

优化阅读 | 精简信息

发件人: Tao Wang<wz1939@163.com>

收件人: 我<shanchan2019@163.com>

时 间: 2022年07月18日 14:20 (星期一)

翻译成中文

想推广找不到途径? 小红书官方全力扶持 立即合作 广告

Dear Dr. Shan,
I agree with the changes to the authors of the manuscript.

Your sincerely,
Tao Wang

At 2022-07-18 12:34:58, "单婵" <shanchan2019@163.com> wrote:

- 隐藏引用文字 -

> Dear authors:
>
> The authors of our article (Long noncoding RNA NONMMUT015745 inhibits doxorubicin-mediated cardiomyocyte apoptosis by regulating Rab2A-p53 axis, CDDISCOVERY-22-3732R1) have been changed. If you confirm and agree to these changes, please reply me via email.
>
> Original authors: Hongjing Cai&,Chan Shan&*, Jie Ju,Tao Wang, Xinzhe Chen, Kai Wang, Fei Wang, Xue Yu, Shaocong Wang, Yin Wang*, Peifeng Li* (& Cai and Shan contributed equally to this work. The corresponding authors are Yin Wang, Chan Shan and Peifeng Li.)
>
> The revised authors: Hongjing Cai&, Pengchao Tian&, Jie Ju,Tao Wang, Xinzhe Chen, Kai Wang, Fei Wang, Xue Yu, Shaocong Wang, Yin Wang, Chan Shan*, Peifeng Li* (& Cai and Tian contributed equally to this work.The corresponding authors are Chan Shan and Peifeng Li.)
>
> Looking forward to receiving your reply.
>
> Yours sincerely,
> Chan Shan

5. Xinzhe Chen

Re: Please confirm the changes of the authors

精简信息

发件人: Xinzhe Chen<sdchenxinzhe@163.com>

收件人: 我<shanchan2019@163.com>

时 间: 2022年07月18日 13:40 (星期一)

翻译成中文

【小红书】如何提高企业订单? 立即合作 广告

Dear Dr. Shan,
I agree to the changes of the authors.

Your sincerely,
Xinzhe Chen

---- Replied Message ----

From 单婵<shanchan2019@163.com>

Date 07/18/2022 13:34

To caihjing81<caihjing81@126.com>,
pchaotian722<pchaotian722@yeah.net>,
JuJiee<jujiee@126.com>,
wz1939<wz1939@163.com>,
sdchenxinzhe<sdchenxinzhe@163.com>,
13592132310<13592132310@163.com>,
wangfei125801<wangfei125801@163.com>,
1367016099<1367016099@qq.com>,
1084499622<1084499622@qq.com>,
wangyin<wangyin@sibs.ac.cn>,
peifli<peifli@qdu.edu.cn>,
我<shanchan2019@163.com>

Subject Please confirm the changes of the authors

Dear authors:

The authors of our article (Long noncoding RNA NONMMUT015745 inhibits doxorubicin-mediated cardiomyocyte apoptosis by regulating Rab2A-p53 axis, CDDISCOVERY-22-3732R1) have been changed. If you confirm and agree to these changes, please reply me via email.

Original authors: Hongjing Cai&,Chan Shan&*, Jie Ju,Tao Wang, Xinzhe Chen, Kai Wang, Fei Wang, Xue Yu, Shaocong Wang, Yin Wang*, Peifeng Li* (& Cai and Shan contributed equally to this work. The corresponding authors are Yin Wang, Chan Shan and Peifeng Li.)

The revised authors: Hongjing Cai&, Pengchao Tian&, Jie Ju,Tao Wang, Xinzhe Chen, Kai Wang, Fei Wang, Xue Yu, Shaocong Wang, Yin Wang, Chan Shan*, Peifeng Li* (& Cai and Tian contributed equally to this work.The corresponding authors are Chan Shan and Peifeng Li.)

Looking forward to receiving your reply.

Yours sincerely,
Chan Shan

6. Kai Wang

Re:Please confirm the changes of the authors 优化阅读 | 精简信息

发件人: Kai Wang<13592132310@163.com>

收件人: 我<shanchan2019@163.com>

时 间: 2022年07月18日 15:13 (星期一)

翻译成中文

【小红书】如何提高企业订单? 立即合作 广告

Dear Dr. Shan,
I agree to the changes of the authors.

Your sincerely,
Kai Wang

At 2022-07-18 13:34:58, "单婵" <shanchan2019@163.com> wrote:

- 隐藏引用文字 -

> Dear authors:
>
> The authors of our article (Long noncoding RNA NONMMUT015745 inhibits doxorubicin-mediated cardiomyocyte apoptosis by regulating Rab2A-p53 axis, CDDISCOVERY-22-3732R1) have been changed. If you confirm and agree to these changes, please reply me via email.
>
> Original authors: Hongjing Cai&,Chan Shan&*, Jie Ju,Tao Wang, Xinzhe Chen, Kai Wang, Fei Wang, Xue Yu, Shaocong Wang, Yin Wang*, Peifeng Li* (& Cai and Shan contributed equally to this work. The corresponding authors are Yin Wang, Chan Shan and Peifeng Li.)
>
> The revised authors: Hongjing Cai&, Pengchao Tian&, Jie Ju,Tao Wang, Xinzhe Chen, Kai Wang, Fei Wang, Xue Yu, Shaocong Wang, Yin Wang, Chan Shan*, Peifeng Li* (& Cai and Tian contributed equally to this work.The corresponding authors are Chan Shan and Peifeng Li.)
>
> Looking forward to receiving your reply.
>
> Yours sincerely,
> Chan Shan

7. Fei Wang

回复：Please confirm the changes of the authors

优化阅读 | 精简信息

发件人：Fei Wang<wangfei125801@163.com>

收件人：我<shanchan2019@163.com>

时 间：2022年07月18日 13:42 (星期一)

翻译成中文

【小红书】如何提高企业订单？ 立即合作 广告

Dear Dr. Shan,
I agree to the changes of the authors.

Your sincerely,
Fei Wang

-------- 原始邮件 --------
发件人：单婵 <shanchan2019@163.com>
日期：2022年7月18日周一 13:34
收件人：caihjing81@126.com, pchaotian722@yeah.net, JuJiee@126.com, wz1939@163.com, sdchenxinzhe@163.com, 13592132310@163.com, wangfei125801@163.com, 1367016099@qq.com, 1084499622@qq.com, wangyin@sibs.ac.cn, peifli@qdu.edu.cn, 我 <shanchan2019@163.com>
主 题：Please confirm the changes of the authors

Dear authors:

The authors of our article (Long noncoding RNA NONMMUT015745 inhibits doxorubicin-mediated cardiomyocyte apoptosis by regulating Rab2A-p53 axis, CDDISCOVERY-22-3732R1) have been changed. If you confirm and agree to these changes, please reply me via email.

Original authors：Hongjing Cai&,Chan Shan&*, Jie Ju,Tao Wang, Xinzhe Chen, Kai Wang, Fei Wang, Xue Yu, Shaocong Wang, Yin Wang*, Peifeng Li* (& Cai and Shan contributed equally to this work. The corresponding authors are Yin Wang, Chan Shan and Peifeng Li.)

The revised authors：Hongjing Cai&, Pengchao Tian&, Jie Ju,Tao Wang, Xinzhe Chen, Kai Wang, Fei Wang, Xue Yu, Shaocong Wang, Yin Wang, Chan Shan*, Peifeng Li* (& Cai and Tian contributed equally to this work.The corresponding authors are Chan Shan and Peifeng Li.)

Looking forward to receiving your reply.

Yours sincerely,
Chan Shan

8. Xue Yu

回复：Please confirm the changes of the authors

优化阅读 | 精简信息

发件人：Xue Yu<1367016099@qq.com>

收件人：我<shanchan2019@163.com>

时　间：2022年07月18日 16:09 (星期一)

【小红书】如何提高企业订单？ 立即合作

Dear Dr. Shan,

I agree to the changes of the authors.

Your sincerely,
Xue Yu

------------------ 原始邮件 ------------------

发件人: "单婵" <shanchan2019@163.com>;
发送时间: 2022年7月18日(星期一) 中午1:34
收件人: "caihjing81"<caihjing81@126.com>;"pchaotian722"<pchaotian722@yeah.net>;"JuJiee"<JuJiee@126.com>;"wz1939"<wz1939@163.com>;"sdchenxinzhe"<sdchenxinzhe@163.com>;"13592132310"<13592132310@163.com>;"wangfei125801"<wangfei125801@163.com>;"我是雪雪呀~"<1367016099@qq.com>;"1084499622"<1084499622@qq.com>;"wangyin"<wangyin@sibs.ac.cn>;"peifli"<peifli@qdu.edu.cn>;"我"<shanchan2019@163.com>;
主题: Please confirm the changes of the authors

Dear authors:

The authors of our article (Long noncoding RNA NONMMUT015745 inhibits doxorubicin-mediated cardiomyocyte apoptosis by regulating Rab2A-p53 axis, CDDISCOVERY-22-3732R1) have been changed. If you confirm and agree to these changes, please reply me via email.

Original authors：Hongjing Cai&,Chan Shan&*, Jie Ju,Tao Wang, Xinzhe Chen, Kai Wang, Fei Wang, Xue Yu, Shaocong Wang, Yin Wang*, Peifeng Li*（& Cai and Shan contributed equally to this work. The corresponding authors are Yin Wang, Chan Shan and Peifeng Li.)

The revised authors：Hongjing Cai&, Pengchao Tian&, Jie Ju,Tao Wang, Xinzhe Chen, Kai Wang, Fei Wang, Xue Yu, Shaocong Wang, Yin Wang, Chan Shan*, Peifeng Li*（& Cai and Tian contributed equally to this work.The corresponding authors are Chan Shan and Peifeng Li.)

Looking forward to receiving your reply.

Yours sincerely,
Chan Shan

9. Shaocong Wang

回复：Please confirm the changes of the authors

优化阅读 | 精简信息

发件人：Shaocong Wang<1084499622@qq.com>

收件人：我<shanchan2019@163.com>

时 间：2022年07月18日 17:15 (星期一)

【小红书】如何提高企业订单？ 立即合作

Dear Dr. Shan,
I agree to the changes of the authors.

Your sincerely,
Shaocong Wang
- 隐藏引用文字 -

------------------ 原始邮件 ------------------

发件人: "单婵" <shanchan2019@163.com>;
发送时间: 2022年7月18日(星期一) 中午1:34
收件人: "caihjing81"<caihjing81@126.com>;"pchaotian722"<pchaotian722@yeah.net>;"JuJiee"<JuJiee@126.com>;"wz1939"<wz1939@163.com>;"sdchenxinzhe"<sdchenxinzhe@163.com>;"13592132310"<13592132310@163.com>;"wangfei125801"<wangfei125801@163.com>;"1367016099"<1367016099@qq.com>;"王少聪"<1084499622@qq.com>;"wangyin"<wangyin@sibs.ac.cn>;"peifli"<peifli@qdu.edu.cn>;"我"<shanchan2019@163.com>;
主题: Please confirm the changes of the authors

Dear authors:

The authors of our article (Long noncoding RNA NONMMUT015745 inhibits doxorubicin-mediated cardiomyocyte apoptosis by regulating Rab2A-p53 axis, CDDISCOVERY-22-3732R1) have been changed. If you confirm and agree to these changes, please reply me via email.

Original authors：Hongjing Cai&,Chan Shan&*, Jie Ju,Tao Wang, Xinzhe Chen, Kai Wang, Fei Wang, Xue Yu, Shaocong Wang, Yin Wang*, Peifeng Li*（& Cai and Shan contributed equally to this work. The corresponding authors are Yin Wang, Chan Shan and Peifeng Li.）

The revised authors：Hongjing Cai&, Pengchao Tian&, Jie Ju,Tao Wang, Xinzhe Chen, Kai Wang, Fei Wang, Xue Yu, Shaocong Wang, Yin Wang, Chan Shan*, Peifeng Li*（& Cai and Tian contributed equally to this work.The corresponding authors are Chan Shan and Peifeng Li.）

Looking forward to receiving your reply.

Yours sincerely,
Chan Shan

10. Yin Wang

Re:Please confirm the changes of the authors

发件人: Yin Wang <wangyin@sibs.ac.cn>

收件人: 我<shanchan2019@163.com>

抄送人: Hongjing Cai <caihjing81@126.com> Pengchao Tian <pchaotian722@yeah.net> Jie Ju <JuJiee@126.com> Tao Wang <wz1939@163.com> Xinzhe Chen <sdchenxinzhe@163.com> ↓还有5个联系人 保存所有抄送人

时 间: 2022年07月19日 10:59 (星期二)

翻译成中文

【小红书】如何提高企业订单? 立即合作 广告

Dear Dr. Shan,
I agree to the changes of the authors.

Your sincerely,
Yin Wang

发件人: "单婵" <shanchan2019@163.com>
发送日期: 2022-07-18 13:34:58
收件人: caihjing81@126.com, pchaotian722@yeah.net, JuJiee@126.com, wz1939@163.com, sdchenxinzhe@163.com, 13592132310@163.com, wangfei125801@163.com, 1367016099@qq.com, 1084499622@qq.com, wangyin@sibs.ac.cn, peifli@qdu.edu.cn, "我" <shanchan2019@163.com>
抄送人:
主 题: Please confirm the changes of the authors

Dear authors:

The authors of our article (Long noncoding RNA NONMMUT015745 inhibits doxorubicin-mediated cardiomyocyte apoptosis by regulating Rab2A-p53 axis, CDDISCOVERY-22-3732R1) have been changed. If you confirm and agree to these changes, please reply me via email.

Original authors: Hongjing Cai&,Chan Shan&*, Jie Ju,Tao Wang, Xinzhe Chen, Kai Wang, Fei Wang, Xue Yu, Shaocong Wang, Yin Wang*, Peifeng Li* (& Cai and Shan contributed equally to this work. The corresponding authors are Yin Wang, Chan Shan and Peifeng Li.)

The revised authors: Hongjing Cai&, Pengchao Tian&, Jie Ju,Tao Wang, Xinzhe Chen, Kai Wang, Fei Wang, Xue Yu, Shaocong Wang, Yin Wang, Chan Shan*, Peifeng Li* (& Cai and Tian contributed equally to this work.The corresponding authors are Chan Shan and Peifeng Li.)

Looking forward to receiving your reply.

Yours sincerely,
Chan Shan

11. Peifeng Li

Re:Please confirm the changes of the authors

优化阅读 | 精简信息

发件人: Peifeng Li<peifli@qdu.edu.cn>

收件人: 我<shanchan2019@163.com>

时 间: 2022年07月18日 15:55 (星期一)

翻译成中文

【小红书】如何提高企业订单? 立即合作

Dear Dr. Shan,
I agree to the changes of the authors.

Your sincerely,
Peifeng Li

------------- Original -------------

From: "单婵"<shanchan2019@163.com>;
Date: Mon, Jul 18, 2022 12:22 PM
To: "ziji"<caihjing81@126.com>; "pchaotian722"<pchaotian722@yeah.net>; "JuJiee"<JuJiee@126.com>; "wz1939"<wz1939@163.com>; "sdchenxinzhe" <sdchenxinzhe@163.com>; "13592132310"<13592132310@163.com>; "wangfei125801"<wangfei125801@163.com>; "1367016099" <1367016099@qq.com>; "1084499622"<1084499622@qq.com>; "王胤老师"<wangyin@sibs.ac.cn>; "peifli"<peifli@qdu.edu.cn>; "我" <shanchan2019@163.com>;
Subject: Please confirm the changes of the authors

Dear authors:

The authors of our article (Long noncoding RNA NONMMUT015745 inhibits doxorubicin-mediated cardiomyocyte apoptosis by regulating Rab2A-p53 axis, CDDISCOVERY-22-3732R1) have been changed. If you confirm and agree to these changes, please reply me via email.

Original authors: Hongjing Cai&,Chan Shan&*, Jie Ju,Tao Wang, Xinzhe Chen, Kai Wang, Fei Wang, Xue Yu, Shaocong Wang, Yin Wang*, Peifeng Li* (& Cai and Shan contributed equally to this work. The corresponding authors are Yin Wang, Chan Shan and Peifeng Li.)

The revised authors: Hongjing Cai&, Pengchao Tian&,Chan Shan, Jie Ju,Tao Wang, Xinzhe Chen, Kai Wang, Fei Wang, Xue Yu, Shaocong Wang, Yin Wang, Chan Shan*, Peifeng Li* (& Cai and Tian contributed equally to this work.The corresponding authors are Chan Shan and Peifeng Li.)

Looking forward to receiving your reply.

Yours sincerely,
Chan Shan
